# AI Chatbot Anthropomorphism and Consumer Decision-Making: A Dual-Pathway Calibration Model

**DOI:** 10.3390/bs16081269

**Published:** 2026-07-23

**Authors:** Qin Zhang, Firdaus Abdullah

**Affiliations:** 1 Faculty of Business and Management, Universiti Teknologi MARA (UiTM), Shah Alam 40450, Selangor, Malaysia; 2022857222@student.uitm.edu.my; 2 Faculty of Business and Management, Universiti Teknologi MARA (UiTM) Cawangan Sarawak, Kota Samarahan 94300, Sarawak, Malaysia

**Keywords:** anthropomorphism, dual-process theory, metacognitive calibration, regulatory focus, human–AI interaction, social cognition

## Abstract

Anthropomorphism has shown inconsistent effects on human judgment, yet the underlying cognitive mechanisms remain underspecified. This research develops and tests a Dual-Pathway Calibration Model (DPCM) integrating dual-process theory, construal level theory, metacognition theory, and regulatory focus theory to explain how anthropomorphic cues influence cognitive processing in human–artificial intelligence (AI) interaction. Three experiments (N = 832) manipulated anthropomorphism and cue inconsistency, examining metacognitive calibration, regulatory focus, and perceived autonomy as boundary conditions. Results revealed that anthropomorphism activates two parallel pathways: (1) a Social Closeness Pathway engaging System 1 processing through reduced psychological distance and enhanced affective trust (indirect effect = 0.18, 95% CI [0.13, 0.24]) and (2) a Cognitive Evaluation Pathway triggering System 2 processing through perceived uncertainty when cue inconsistency is present (ηp^2^ = 0.06). Metacognitive calibration moderated the AI-reliance–decision-quality relationship (β = 0.17, *p* = 0.007). Regulatory focus moderated pathway activation, with promotion focus strengthening Pathway A (ηp^2^ = 0.15) and prevention focus strengthening Pathway B (ηp^2^ = 0.05). Anthropomorphism exhibited an inverted U-shaped relationship with decision outcomes (quadratic b = −0.05, *p* = 0.001). These findings extend dual-process theory by specifying conditions triggering intuitive versus analytical processing of anthropomorphic agents, contribute to metacognition theory by demonstrating calibration as a critical determinant of AI-assisted judgment quality, and advance regulatory focus theory by showing motivational orientation shapes social cue processing from artificial agents.

## 1. Introduction

Artificial intelligence (AI) has significantly transformed how business organizations engage with consumers. AI-based chatbots are now widely used to provide customer service and personalized services across sectors such as retail, healthcare, and finance (Cheng et al., 2022), and they are increasingly designed with anthropomorphic features to promote consumer engagement (Blut et al., 2021). Industry analyses indicate that by 2025 more than 80% of customer interactions will be handled by AI-based chatbots, making consumers’ responses to these technologies critically important from both academic and managerial perspectives.

Anthropomorphism, defined as the attribution of human qualities to non-human entities, has become a dominant design paradigm for AI chatbots (Sheehan et al., 2020). A comprehensive meta-analysis by Blut et al. (2021) integrated findings across robots, chatbots, and other AI technologies and showed that anthropomorphism increases consumer acceptance. Related work indicates that anthropomorphic chatbots raise consumer trust through perceived warmth and competence while reducing switching intentions (Cheng et al., 2022), strengthen perceived personalization and willingness to pay premiums in conversational commerce (Sidlauskiene et al., 2023), and enhance customer satisfaction through enjoyment, attitude, and trust (Klein & Martinez, 2023).

Nevertheless, the relationship between chatbot anthropomorphism and consumers is not uniformly positive. Emerging research indicates that anthropomorphism can act as a “double-edged sword” that, under certain conditions, elicits negative consumer responses (Crolic et al., 2022), echoing service-robot findings that humanoid design can trigger mixed reactions (Mende et al., 2019). Crolic et al. (2022) showed that when consumers are angry, chatbot anthropomorphism reduces satisfaction and purchase intentions, contradicting the assumption that humanlike cues are invariably beneficial. Over-humanization can also backfire: Song and Shin (2024) found that overly humanlike virtual agents in e-commerce induce discomfort, undermining perceived credibility and technology acceptance. Similarly, Kim et al. (2025) advanced the construct of “cognitive uncanniness,” whereby users attribute negative humanlike intentions to agents with sophisticated cognitive abilities, raising concerns about information security and exploitation.

User autonomy is another important dimension in evaluating human–AI conversational interactions. Studying user behavior toward the visual attributes and activation mechanisms of virtual assistants, Pizzi et al. (2021) found that humanlike interfaces reduce defensive psychological responses, which in some cases can compromise satisfaction with decision-making. Z. Lu et al. (2024) extended this line of work by examining how humanized conversational design affects user migration after service disruption, with remedial performance projection as a mediator. Together, these studies suggest that the impact of humanized interface design is conditional and warrants further investigation.

Although research on humanization in conversational AI has advanced substantially, three theoretically consequential problems remain unresolved, and none can be addressed by extending any single existing account. First, dominant unidirectional trust models predict a monotonic, generally positive effect of anthropomorphism on reliance and outcomes; such models cannot, in principle, reproduce the well-documented curvilinear and double-edged findings in which humanlike cues sometimes help and sometimes harm. Second, dual-process theory establishes that consumers shift between intuitive and analytical processing but, in its general form, does not specify what triggers the switch in the case of an artificial social agent—why and when an anthropomorphic chatbot moves a consumer from heuristic acceptance to effortful scrutiny—nor how AI dependency feeds forward into decision quality. Third, existing trust-calibration work does not explain the heterogeneity in outcomes among consumers who rely on AI to an equal degree, nor does it connect it to stable individual-difference factors that predict which route consumers take. These are three faces of a single missing construct: a model that jointly specifies the architecture of processing, the mechanism that routes consumers into each mode, the gate that determines whether reliance helps or harms, and the conditions under which these relationships hold. The Dual-Pathway Calibration Model (DPCM) is advanced to fill this integrated gap, consolidating rather than proliferating theory by importing each component only to the minimal extent required to close a gap the others leave open.

Recent studies have begun to examine the effect of regulatory focus on user interaction with AI chatbots. Khan et al. (2024) show that consumers with a promotion orientation can mitigate the negative effect of chatbots on consumer decisions, and Fu et al. (2026) examined regulatory focus as a boundary condition for chatbot humanization and willingness to share information. These findings align with broader work on regulatory focus in consumer behavior (Werth & Foerster, 2007), which holds that promotion-oriented consumers pursue advancement through eager strategies while prevention-oriented consumers pursue safety through vigilant strategies. However, an integrated conceptual model is still missing.

To fill this knowledge gap, this research proposes the DPCM, which integrates four theories: dual-process theory, construal level theory, metacognition theory, and regulatory focus theory. In the model, chatbot anthropomorphism can influence consumer decision-making through two paths. Path A, the “Social Closeness Pathway,” rests on reduced psychological distance and affective trust, which trigger heuristic-based reliance on AI recommendations. Path B, the “Cognitive Evaluation Pathway,” is triggered when high anthropomorphism is paired with cue inconsistency, inducing perceived uncertainty and cognitive appraisal of AI recommendations. Crucially, the model proposes metacognitive calibration as a key moderator determining whether AI-based reliance yields better or worse decision quality.

The present research makes a single, integrative theoretical contribution—the Dual-Pathway Calibration Model—best understood through four interlocking facets rather than as four separate claims. The unifying contribution is to recast consumer responses to chatbot anthropomorphism as a routed, gated, and bounded process rather than a unidirectional trust effect. Its first facet replaces the prevailing unidirectional model with bifurcated processing channels, explaining the mixed empirical record that monotonic trust models cannot. Its second introduces metacognitive calibration as the gate that determines whether reliance translates into better or worse decisions, extending metacognition theory into the consumer-AI reliance domain and accounting for outcome heterogeneity with equal reliance. Its third formalizes the curvilinear, inverted-U relationship between anthropomorphism and intuitive engagement, identifying an optimal anthropomorphization threshold. Its fourth specifies motivational and autonomy-based boundary conditions that shape pathway activation and the reliance-to-outcome link. The novelty of the DPCM lies not in any single element, several of which have antecedents in the literature, but in their integration: prior work has offered unidirectional trust models, single-mode dual-process descriptions, and standalone calibration constructs in isolation, whereas the DPCM brings architecture, mechanism, gate, and boundary conditions together into one falsifiable framework tested across three experiments.

From a practical point of view, this research provides evidence-based advice to those involved in AI system design and marketing. The research indicates that moderate levels of anthropomorphism, rather than maximum humanization, may produce the best outcomes for consumers. In addition, by identifying regulatory focus as a boundary condition, this research provides personalized strategies for designing chatbots that are appropriate for different segments of consumers. Finally, the focus on metacognitive calibration underscores the need to develop AI interfaces that enhance consumers’ capacity to judge the reliability of AI recommendations, thereby improving the effectiveness of human–AI collaboration.

## 2. Theoretical Background and Hypotheses

### 2.1. AI Chatbot Anthropomorphism

The concept of humanization is defined as the cognitive process whereby people attribute anthropomorphic qualities to artificial entities. In the context of artificial intelligence conversational systems, the concept of humanization encompasses morphological characteristics, patterns of linguistic interaction, emotional expressiveness, and cognitive qualities. In the context of artificial intelligence conversational systems, there are a number of humanization techniques used to generate personalized naming systems, visual representations, patterns of conversation, humor integration, and personal disclosure systems.

The responses to chatbot humanization carry both positive and negative valence. Positive effects include increased warmth and competence, social presence, emotional connection, and satisfaction, and meta-analytic evidence confirms that anthropomorphism generally enhances outcomes across human–robot and human–AI interaction (Roesler et al., 2021). These effects are often explained through the Computers Are Social Actors (CASA) perspective, which assumes that people spontaneously apply social norms to computer-based agents.

However, anthropomorphism can also have undesirable effects under certain circumstances. Too much humanization may cause the uncanny valley effect, in which non-human objects that are too similar to humans may cause uneasiness. Chatbots that are too anthropomorphic may also create high expectations that, when not met, may cause higher disappointment and dissatisfaction. Another potential disadvantage that may come as a result of the issue of privacy is that consumers may view these anthropomorphic agents as having autonomous power to misuse information. The different research findings have shown that the relationship between anthropomorphism and consumer outcomes is not a linear function, but it is based on theoretical frameworks that have room for facilitating as well as inhibitory factors.

### 2.2. Dual-Process Theory and Information Processing

Bifurcated frameworks of processing are the basic paradigms of human judgment and decision-making, as emphasized by the cognitive and social psychological domains of study. An extensive overview of dual system and processing frameworks of consumer psychology is provided by Samson and Voyer (2012), which emphasizes the difference between Type 1 processing, characterized as “spontaneous, effortless, and pattern-driven,” and Type 2 processing, characterized as “systematic, effortful, and principle-driven.” Type 1 operations are conducted rapidly and smoothly through cognitive “shortcuts” and “affective mechanisms,” while Type 2 operations are conducted methodically and consciously through the evaluation of information by consumers.

The latest developments in the theoretical underpinning of the bifurcated processing model have made it possible to expand the domain of the bifurcated processing model to the realm of artificial intelligence. The research conducted by Brady et al. (2025) is based on the dual mechanisms of the two-system approach in the realm of artificial intelligence and large language models. The extension of the bifurcated processing model is very significant for the information processing mechanism of the AI-based consumer-facing conversational agents.

The dual-process theory in the context of human–AI interaction suggests that the anthropomorphic chatbot is more likely to influence human decisions through System 1 processing based on the perception of social presence that would evoke anthropomorphic responses. On the other hand, the presence of cues that are associated with artificiality or expectation violation is more likely to trigger System 2 processing for the evaluation of AI recommendations. This dual-process theory is the underpinning for the proposed model.

### 2.3. The Dual-Pathway Calibration Model (DPCM)

Based on the theoretical underpinnings discussed above, the research proposes a Dual-Pathway Calibration Model (DPCM) that identifies the manner in which chatbot anthropomorphism influences consumer decision-making in two unique but equivalent paths. The proposed model argues that anthropomorphism simultaneously triggers a Social Closeness Pathway (Pathway A) that leads to a greater dependence on heuristic-based AI and a Cognitive Evaluation Pathway (Pathway B) that triggers the analytical evaluation of AI suggestions. The extent to which each of these processes occurs also impacts the nature and quality of consumer decisions, and metacognitive calibration is a significant moderator in the transfer of reliance on AI to decision outcomes.

#### 2.3.1. Pathway A: Social Closeness Pathway

The Social Closeness Pathway is built on the basis of construal level theory (CLT), which claims that psychological distance is the fundamental factor that influences the mental representation of objects, events, and entities from a psychological viewpoint (Trope & Liberman, 2010). Trope and Liberman demonstrated that psychological distance is a multidimensional construct that includes time, space, social, and hypothetical distances, where the increase in psychological distance is associated with higher-level construals and the decrease in psychological distance is associated with lower-level representations. In addition, the results of the study by Liberman and Trope (2008) further showed that the experience of transcending the immediate “here and now” was an important component of human cognition, and that psychological distance was an important determinant of judgment and decision processes.

With regard to the interaction between humans and AI, Kirshner (2025) examined the relationship between psychological distance and aversion to algorithms. The results showed that lower levels of psychological distance between consumers and AI advisors are positively related to the acceptance of algorithm-based recommendations by consumers, especially when the AI advisor expresses high confidence. This study shows that factors that reduce the perceived distance between consumers and AI agents can have a positive effect on the acceptance of AI-based recommendations. The anthropomorphic design of chatbots, which ascribes human-like attributes to AI agents, minimizes the distance between consumers.

In their study of user preferences for humanized versus mechanized AI interface agents in pre-purchase contexts, M. Zhang et al. (2025) identified contextual variation in humanization preferences and their links to dispositional factors, supporting the claim that anthropomorphism-induced interpersonal affinity promotes user engagement and consumption. Weidlich et al. (2024) offered a mental-representation model linking interpersonal presence to proximity, explaining how presence reduces remoteness and enhances the positivity of digitally mediated interactions. Consistent with this pathway, Jin and Youn (2023) found that social presence predicts chatbot continuance intention, and Park et al. (2024) demonstrated a serial mediation of psychological distance and trust on compliance intention in chatbot interaction, directly paralleling the distance-to-trust sequence proposed here.

The Social Closeness Pathway follows a sequential pattern in which anthropomorphism reduces psychological distance, which in turn increases affective trust, a type of trust that is emotional and relationship-oriented, involving feelings of security, being cared for, and relatedness. Affective trust increases heuristic-based trust in AI recommendations, in which consumers comply with chatbots’ recommendations with minimal cognitive processing. This pathway follows a System 1 processing style, which is quick, intuitive, and relies on social and emotional cues.

The ordering from psychological distance to affective trust is not arbitrary but is dictated by construal level theory: psychological distance is a determinant of construal and of the affective proximity on which warmth-based trust rests, so reduced distance is theoretically prior to, and a cause of, affective trust (Trope & Liberman, 2010). We nonetheless acknowledge that alternative causal sequences are conceivable; for example, an initial affective reaction to humanlike cues could retroactively reduce perceived distance, or the two could be reciprocally constituted. Because our design measures both constructs, we treat the serial ordering as an empirical question and test the hypothesized distance-to-trust path against these alternatives rather than assuming it; the serial-mediation specification is adopted because it is the sequence that CLT predicts a priori.

**Hypothesis** **1** **(H1).** *Chatbot anthropomorphism positively influences consumers’ heuristic-based AI reliance through the serial mediation of (a) reduced psychological distance and (b) enhanced affective trust.*

#### 2.3.2. Pathway B: Cognitive Evaluation Pathway

Whereas Pathway A reflects the facilitating effects of anthropomorphism, the Cognitive Evaluation Pathway explains the circumstances under which anthropomorphism might elicit more cognitively demanding processing. This pathway is engaged when high anthropomorphism combines with cue inconsistency—in other words, when the anthropomorphic appearance or communication style of a chatbot clashes with its actual performance or responses.

The theoretical foundation of this channel is based on the concept of the “uncanny valley of mind,” which is based on the uncanny valley effect, including mental attributes as well as physical attributes. This is because, based on the assessment of the consumer regarding the high human-like quality of the chatbot, as well as the discrepancies that indicate the artificial nature of the chatbot, such as the inappropriate response or lack of comprehension, there is a significant increase in the overall level of uncertainty that the consumer feels regarding the chatbot, causing the consumer to change their processing style from heuristic to analytical based on the use of System 2 to evaluate the trustworthiness of the AI agent.

It should be noted that the activation of the process of analytical evaluation does not necessarily lead to negative outcomes. Rather, it can be considered a more controlled process of information processing, which can lead to more informed decisions of reliance, accepting or declining the recommendations based on the presence of issues related to the quality of the information. The key difference is that, unlike in Pathway A, there is a conscious process of assessment in Pathway B.

To ground the uncertainty mechanism in established theory, we locate it within expectancy violations theory (Burgoon, 1993): a highly anthropomorphic agent activates strong humanlike expectations, and cue inconsistency constitutes a violation of those expectations, which is the proximal source of the heightened uncertainty we predict. This framing also resolves a potential conceptual overlap between uncertainty and analytical evaluation. We define the two as distinct in kind: perceived uncertainty is an epistemic state, a subjective sense that the agent’s behavior is unpredictable or that the recommendation cannot be confidently accepted, whereas analytical evaluation is the effortful process (System 2 engagement) that the uncertainty state motivates. Uncertainty is thus the trigger and analytical evaluation the response; they are causally ordered rather than redundant, and the hypothesis specifies uncertainty as the mediator that converts an expectancy violation into a shift in processing mode.

**Hypothesis** **2** **(H2).** *When high anthropomorphism is accompanied by cue inconsistency, consumers experience increased perceived uncertainty, which activates analytical evaluation of AI recommendations.*

### 2.4. Metacognitive Calibration as Moderator

Metacognition, or the ability to control one’s cognitive processes, is vital in determining the impact of reliance on AI on the quality of decisions made. In this respect, Tankelevitch et al. (2024) investigated the metacognitive demands and opportunities of generative AI. From their analysis, there are several challenges that an individual using AI faces in making an accurate judgment about the reliability of the information provided by the AI. This analysis shows that for effective collaboration between humans and AI, there is a need to develop metacognitive capabilities.

Fleming (2024) gave a comprehensive overview of metacognition and confidence, which integrated the findings related to the ways in which people monitor their own knowledge states and regulate their behaviors based on these judgments. Metacognitive calibration is the term used to describe the relation between confidence and accuracy; well-calibrated people tend to have high confidence when they are correct and low confidence when they are likely to be wrong. In terms of AI-assisted decision-making, calibration refers to consumers’ ability to gauge whether or not AI recommendations are trustworthy and should be followed or are untrustworthy and should not be followed.

The relevance of calibration in human–AI interaction has also been validated through empirical studies. Rechkemmer and Yin (2022) investigated the role of multiple performance metrics on the development of trust in machine learning models. The results showed that the presentation of accuracy and confidence metrics to the user could help in the development of more accurate calibration. Y. Zhang et al. (2020) examined the role of confidence information and explanations on the development of more accurate calibration. Steyvers and Kumar (2024) identified the following three basic challenges to AI-based decision-making: understanding the capabilities of AI, proper calibration of the level of trust, and the integration of AI-based recommendations with human judgment. Their study emphasized the significance of the proper calibration of the level of trust in AI-led systems to avoid suboptimal decision outcomes.

Algorithm aversion is a form of miscalibration that is characterized by over-skepticism toward algorithm recommendations. Jussupow et al. (2020) conducted an extensive literature survey on algorithm aversion and determined the factors that make consumers algorithm-averse, even when the algorithm recommendations are better than human judgments. It is important to understand the antecedents and consequences of algorithm aversion to design the right AI for collaboration with humans.

In the new DPCM, metacognitive calibration plays a major role as a moderator between the usage of AI and decision quality. When consumers exhibit good metacognitive calibration, increased usage of AI recommendations will lead to improved decision quality because consumers are likely to behave according to good advice from AI. On the other hand, poor metacognitive calibration exhibited by consumers, such as through over- or under-confidence with AI recommendations, may cause failure or even negative decision outcomes.

It is important to distinguish metacognitive calibration from two adjacent constructs. Confidence is a first-order judgment about a single decision (how sure one feels); trust calibration concerns the appropriate matching of trust to a system’s overall reliability. Metacognitive calibration, by contrast, is a second-order, discriminative capacity: the accuracy with which a consumer’s sense of when to accept versus question a recommendation tracks the actual quality of that recommendation (Fleming, 2024; Steyvers & Kumar, 2024). A consumer can be highly confident yet poorly calibrated or appropriately trusting of the system on average yet unable to discriminate good from bad outputs case by case. The mechanism linking calibration to decision quality is selective acceptance: well-calibrated consumers accept good recommendations and override poor ones, so the same level of reliance yields higher decision quality, which is why calibration moderates, rather than merely adds to, the reliance–quality relationship.

**Hypothesis** **3** **(H3).** *Metacognitive calibration moderates the relationship between AI reliance and decision quality, such that the positive effect of AI reliance on decision quality is stronger (weaker) when calibration is high (low).*

### 2.5. Regulatory Focus as Boundary Condition

According to the theory of regulatory focus, there exist two self-regulatory systems that aid in achieving set goals. The two self-regulatory systems include advancement orientation and security orientation (Scholer et al., 2014). Advancement-oriented individuals are concerned with aspirational outcomes. These individuals emphasize the importance of achievement and development. Advancement-oriented individuals employ eager strategies to achieve their desired outcome. Security-oriented individuals are concerned with security and stability. These individuals employ cautious strategies to avoid negative outcomes. The two self-regulatory systems affect various aspects of consumer behavior.

Scholer et al. (2014) discovered that regulatory orientation has an impact on risk preference behaviors. The study demonstrated that subjects with a security orientation have more risk-taking behaviors when they rate themselves as being below protective standards. This illustrates the contextual variability of regulatory focus effects. In their examination of the persuasive effects of regulatory congruence, Lee and Higgins (2009) demonstrated that being congruent with an individual’s regulatory condition facilitates easier cognitive processing and more favorable evaluation. Regulatory congruence generates subjective feelings of appropriateness, thus increasing levels of engagement.

In the case of human–AI interaction, regulatory focus can be considered a boundary condition, which influences the relative activation of the dual pathways. For example, consumers with a promotion-focused mindset, who are oriented towards gains and are eager to seize opportunities, may be more inclined to the social closeness cues offered by anthropomorphic chatbots, thus leading to a higher activation of Pathway A. Their concern with potential benefits rather than risks may help create affective trust and heuristic-based trust. On the other hand, prevention-focused consumers, with their concern with safety and watchfulness towards potential risks, may be more responsive to inconsistency cues and more inclined to engage in analytical evaluation as described in Pathway B. Their concern with mistakes may make them more inclined to scrutinize recommendations made by AI.

The prediction is not reducible to promotion-focused consumers simply showing greater general technology acceptance; it is specific to the content of anthropomorphic cues. Promotion focus orients attention toward gains, advancement, and the eager pursuit of positive end-states, which makes the affiliative, warmth-signaling cues of an anthropomorphic agent especially motivationally resonant and thereby amplifies the affective, intuitive Social Closeness Pathway (Scholer et al., 2014; Lee & Higgins, 2009). Prevention focus, in contrast, orients attention toward security, responsibility, and the vigilant avoidance of errors and losses; this vigilance disposes prevention-focused consumers toward effortful scrutiny of whether a recommendation is correct, aligning them with the analytical Cognitive Evaluation Pathway. The two foci therefore predict differential route activation (which mechanism dominates), not merely differential levels of overall acceptance, a distinction our pathway-specific analyses are designed to detect.

**Hypothesis** **4** **(H4).** *Regulatory focus moderates the relative activation of the dual pathways, such that (a) promotion-focused consumers exhibit stronger activation of the Social Closeness Pathway (Pathway A), while (b) prevention-focused consumers exhibit stronger activation of the Cognitive Evaluation Pathway (Pathway B).*

### 2.6. Perceived Autonomy as Boundary Condition

Self-directed agency, which refers to the subjective experience of self-regulatory control and self-governance of behavioral choices by individuals, might be a fundamental psychological precursor that has the potential to impact the way consumers behave with AI-assisted decision-making systems. In their study of autonomy concerns related to the adoption of highly autonomous artificial intelligence technologies, the research by Frank and Otterbring (2024) identified that threats to self-direction can result in oppositional behavioral responses towards AI technologies among those for whom self-regulatory control is of significant personal importance.

In the context of the exploration of perceived human agency and defensive behavior in AI-mediated interactions, Sankaran et al. (2021) demonstrated the occurrence of reactance, defined as “a motivational state characterized by resistance and counter-arguing,” when consumers perceive the intrusiveness and coerciveness of AI-recommended choices. In the same vein as the above studies, W. Lu (2024) investigated the ethical implications of personalization in AI-mediated decision-making processes and argued that individual autonomy has to be considered an ethical imperative.

André et al. (2018) gave a broader perspective on consumer choice and autonomy in the context of the digital age of artificial intelligence and big data, specifically with regard to the role of artificial-intelligence-based systems of personalization and recommendations on consumer autonomy. André et al. explained that artificial intelligence is not only capable of limiting the cognitive burden of consumer choices but is also capable of limiting the scope of consumer choices, of which the consumer is unaware. An empirical study by Fan and Liu (2022) was carried out to examine the impact of algorithmic decision autonomy on consumer purchase behavior. The study revealed that the autonomy of AI algorithms influences the satisfaction of consumers in AI-mediated transactions.

Under the context of the proposed theoretical model, the perception of autonomy is considered to be a contextual moderator that would influence the relationship between reliance on AI systems and corresponding decision satisfaction outcomes. Therefore, in cases of high perceived autonomy in which the individual is voluntarily in control of the decision process while still possessing discretionary freedom to agree or reject decisions made by AI systems, there is a positive relationship that exists between reliance on AI systems and corresponding decision satisfaction.

We specify perceived autonomy as a moderator rather than a direct antecedent because, theoretically, autonomy does not by itself create satisfaction; rather, it governs how a given level of reliance is translated into satisfaction. When perceived autonomy is high, relying on AI is experienced as a self-endorsed choice, so reliance converts readily into satisfaction; when perceived autonomy is low, the same reliance can elicit psychological reactance and a sense of constrained agency, attenuating or even reversing its satisfying effect (Frank & Otterbring, 2024; Sankaran et al., 2021). Autonomy thus operates on the slope of the reliance-to-satisfaction relationship, not on its intercept. Modeling it as a main-effect antecedent would mis-specify its role and obscure the reactance mechanism through which it actually operates, which is why a moderation specification is theoretically required.

**Hypothesis** **5** **(H5).** *Perceived autonomy moderates the relationship between AI reliance and decision satisfaction, such that the positive effect of AI reliance on satisfaction is stronger (weaker) when perceived autonomy is high (low).*

### 2.7. Nonlinear Effect of Anthropomorphism (Exploratory)

The preceding hypotheses emphasize mechanisms and boundary conditions that moderate the anthropomorphism effect. A final exploratory research question pertains to the functional form of the relationship between anthropomorphism and outcomes. Note that Pathway A and Pathway B are competing forces that may be differentially activated at different levels of anthropomorphism. Thus, the net effect may not be monotonic.

At low levels of anthropomorphism, AI agents may be viewed as unemotional, impersonal tools that do not engage consumers at an emotional level, thereby not strongly activating Pathway A. At moderate levels of anthropomorphism, warmth and social presence increase, thereby strongly activating Pathway A with favorable outcomes without evoking concerns about the uncanny valley or cue inconsistency. However, when anthropomorphism reaches high levels, the probability of expectation violations and inconsistencies increases, which activates Pathway B, leading to possible negative reactions that cancel out the effects of social closeness.

This suggests that the relationship between anthropomorphism and decision outcomes should be inverted U-shaped. While the current hypothesis is offered as speculative in the absence of direct empirical evidence, it combines the logic of the dual pathway model to offer a specific prediction about the form of the relationship.

The inverted-U follows from the superposition of two opposing forces operating on the same anthropomorphism continuum, a competing-forces logic that specifies the inverted-U over alternative nonlinear forms. As anthropomorphism rises from low to moderate, social-presence and affective-trust benefits accumulate and the Social Closeness Pathway (Pathway A) strengthens, producing the ascending limb. As it rises further, expectancy violations and cue inconsistencies become more likely, increasingly activating the uncertainty-driven Cognitive Evaluation Pathway (Pathway B) and eroding intuitive engagement, producing the descending limb. Because both forces vary continuously and are oppositely signed—with the benefit force saturating while the cost force keeps growing—a single-peaked, concave (inverted-U) function is the form uniquely consistent with these mechanisms, rather than a threshold, J-shaped, or monotonic form.

**Hypothesis** **6** **(H6,** **Exploratory).** *The relationship between chatbot anthropomorphism and consumer decision outcomes (quality and satisfaction) follows an inverted U-shaped pattern, such that moderate levels of anthropomorphism yield optimal outcomes.*

### 2.8. The Integrative Logic of the DPCM

The four theories underpinning the DPCM are not assembled additively; each occupies a distinct explanatory role, and the framework’s coherence derives from the way they constrain one another. Dual-process theory supplies the architecture of the model: it specifies that responses to an anthropomorphic agent can be governed by either an intuitive, heuristic mode (System 1) or a deliberate, analytical mode (System 2) and that these modes can be differentially engaged (De Neys, 2014). Dual-process theory is deliberately agnostic about content, however; it describes the existence of two modes but not the substantive mechanism through which a social cue such as anthropomorphism recruits the intuitive mode, nor what determines whether the resulting reliance is beneficial. The remaining three theories supply precisely these missing specifications.

Construal level theory specifies the mechanism of the intuitive pathway. It explains why humanlike cues are not merely liked but psychologically operative: anthropomorphism reduces social and psychological distance, and reduced distance is the established antecedent of more concrete, proximal, affect-laden construal (Trope & Liberman, 2010). CLT thus converts the abstract System 1 route into a testable causal sequence (distance to affective trust to heuristic reliance) that dual-process theory alone cannot generate.

Metacognition theory specifies the evaluative gate that determines the consequences of reliance. Dual-process accounts predict that reliance occurs, but not whether it improves or degrades decisions. Metacognitive calibration, the accuracy with which a consumer discriminates trustworthy from untrustworthy AI output (Fleming, 2024; Steyvers & Kumar, 2024), is the variable that separates adaptive from maladaptive reliance, and it is therefore necessary to explain heterogeneity in decision quality at equal levels of reliance.

Finally, regulatory focus theory and perceived autonomy specify boundary conditions. Regulatory focus (Scholer et al., 2014) governs which pathway is preferentially activated by determining the consumer’s sensitivity to affiliative versus vigilant cues, while perceived autonomy governs the translation of reliance into satisfaction. In short, dual-process theory defines the stage, CLT scripts the intuitive route, metacognition adjudicates outcomes, and regulatory focus and autonomy set the conditions of performance. No single theory can perform all four functions; their integration is therefore structural rather than ornamental.

### 2.9. Competing Theoretical Explanations

Two simpler accounts might be advanced as alternatives to the DPCM, and considering them clarifies the model’s added value. The first is a pure affective-trust (CASA) account, which holds that anthropomorphism elicits social responses and affective trust that monotonically increase reliance and satisfaction. This parsimoniously explains the intuitive pathway, but because it is monotonic, it cannot explain the downturn in consumer responses at high anthropomorphism, offers no mechanism for switching consumers into analytical processing, and is silent on why equal reliance yields unequal decision quality. The second is a pure uncanny valley account, which attributes negative responses at high humanlikeness to discomfort or eeriness; this explains the downturn but not the beneficial intuitive route at moderate anthropomorphism, the individual differences in which route dominates, or the role of calibration in determining outcomes. The DPCM integrates the strengths of both: it accommodates the affective upside (via the Social Closeness Pathway), the high-anthropomorphism downturn (via expectancy-violation-triggered activation of the Cognitive Evaluation Pathway), and the outcome heterogeneity (via the calibration gate) within one framework, and it specifies boundary conditions that these narrower accounts do not address. We therefore position the DPCM as a broader integrative framework rather than a definitive account, valued for its explanatory scope rather than its parsimony.

### 2.10. Theoretical Model Summary

**Figure 1:**
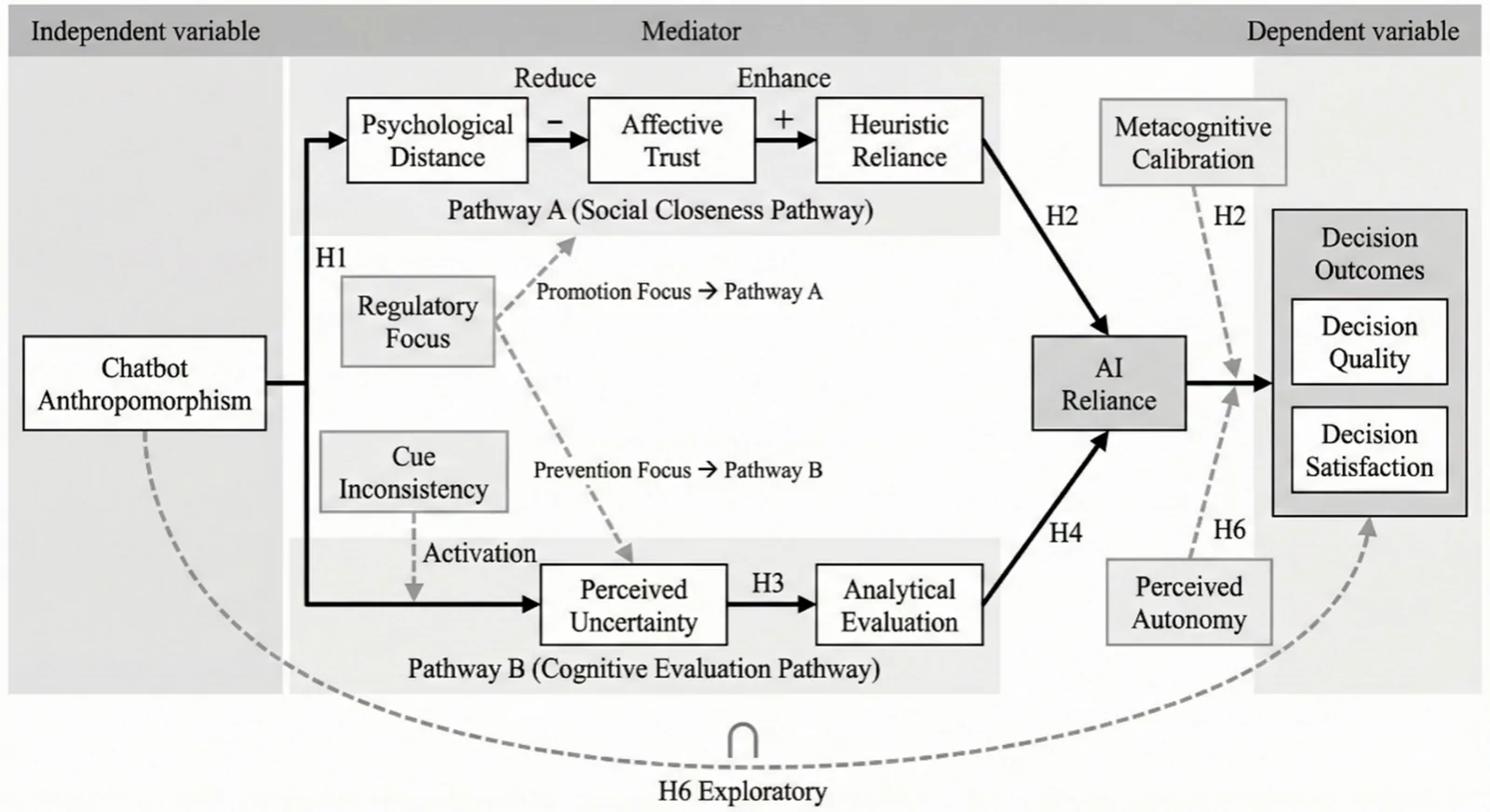
The Dual-Pathway Calibration Model (DPCM)—Conceptual Framework. Solid arrows indicate hypothesized direct paths; dashed arrows indicate moderating effects; the dashed curve at the bottom indicates the exploratory inverted U-shaped relationship (H6).

The Dual-Pathway Calibration Model (DPCM) developed in the current investigation synthesizes several theories to provide a more integrative account of the chatbot anthropomorphism effect on consumer decision-making. The complete conceptual model of the DPCM is presented in Figure 1, and its hypotheses are summarized in Table 1.

As depicted in Figure 1, there are two parallel pathways activated by anthropomorphism: Pathway A—Social Closeness Pathway: the mechanism is based on the decrease in psychological distance and increase in affective trust, which results in heuristic-based reliance on AI; Pathway B—Cognitive Evaluation Pathway: the activation is based on the co-occurrence of anthropomorphism and cue inconsistency, which leads to uncertainty and analytical evaluation. The link between AI reliance and decisions is moderated by metacognitive calibration and perceived autonomy, while the role of regulatory focus is to modulate the activation level of the two pathways. The model also predicts a nonlinear (inverted U-shaped) overall effect of anthropomorphism on decision outcomes.

## 3. Method

### 3.1. Overview of Studies

In order to validate the proposed theoretical framework of the Dual-Pathway Calibration Model (DPCM), a series of three empirical research studies were undertaken. The first research study focused on the fundamental bifurcated processing mechanisms by testing hypotheses H1 and H2. The second research study focused on the conditional effects of metacognitive calibration and regulatory focus orientation, which tested hypotheses H3 and H4. The third research study focused on the role of perceived autonomy as a contextual constraint (H5) while also exploring the possibility of curvilinear relationships between anthropomorphism and decision outcomes (H6). Table 2 provides an integrative synthesis of all three research approaches.

All studies were conducted online using Credamo (www.credamo.com), a well-established Chinese survey platform for behavioral research. Participants were recruited from mainland China and were required to be 18–55 years old with prior AI chatbot experience. The research protocol was approved by the Guangdong Academy of Social Sciences Ethics Committee (Approval No. SMEC-2025-037). All procedures followed the Declaration of Helsinki. Participants provided electronic informed consent and received 8–15 CNY compensation. Data collection occurred between March and June 2025.

**Transparency and open materials.** In the interest of reproducibility, the complete experimental stimuli, full-color screenshots of the chatbot interfaces for each anthropomorphism condition, all manipulation and manipulation-check materials, and the verbatim items for every measured scale are provided in Appendix A, Appendix B and Appendix C and archived on the OSF. The repository additionally contains the pre-registration documents, the expert-benchmark scoring protocols, and the analysis scripts for the study-specific and pooled models.

**Data screening and exclusions.** Prior to analysis, data were screened against pre-registered quality-control criteria. Participants were excluded if they failed one or more attention or instructional manipulation checks, completed the study faster than a pre-specified minimum completion-time threshold (indicative of non-substantive responding), exhibited straight-lining (invariant responses across reverse-keyed items), or failed embedded comprehension checks verifying that they had attended to the scenario and recommendation. Participants who failed one or more of these checks were excluded prior to the final samples reported below (Study 1 N = 286; Study 2 N = 234; Study 3 N = 312), which therefore comprise only respondents meeting all data-quality criteria.

**Randomization.** Assignment to experimental conditions in all studies was performed by computer-generated random allocation implemented through the Credamo platform, using equal-probability allocation to each cell of the design. Condition assignment was concealed from participants, who were unaware of the existence of alternative conditions, and was generated automatically at the point of survey entry; in the multifactor designs, randomization across the orthogonal factors (including the secondary cue-inconsistency factor in Study 2 and the optional calibration module in Study 3) was carried out independently for each factor.

### 3.2. Study 1: Dual-Pathway Mechanism

#### 3.2.1. Participants and Design

The first study employed a factorial experimental design that involved three levels of anthropomorphism (low, medium, high) × two levels of cue inconsistency (absent, present) with independent group assignment. A priori power analysis indicated that a minimum of 251 participants were required to detect effect sizes of f = 0.20 with 80% statistical power. The final sample consisted of 286 participants (M = 31.4 years old, SD = 8.7; 53.1% female; 71.3% held a bachelor’s degree or higher). Participants were randomly assigned to the six experimental conditions, consisting of 45 to 50 participants per condition. Those participants who did not pass the vigilance checks were removed.

#### 3.2.2. Procedure

The participants were presented with a scenario that involved making an investment decision with an amount of 30,000 CNY. Then, the participants interacted with an AI financial advisor chatbot. During the interaction with the chatbot, the participants were assisted in identifying their risk tolerance, investment horizon, and objectives. After that, the participants completed the manipulation check and the dependent measure.

#### 3.2.3. Manipulations

Anthropomorphism was manipulated through visual appearance, identity, and communication style. The low condition featured an abstract AI icon with a functional system label and formal language. The medium condition presented a stylized cartoon avatar with a friendly nickname and conversational tone. The high condition displayed a realistic human photograph with a personal name and warm, empathetic communication including self-disclosure. Manipulation specifications are detailed in Table 3. A pretest (N = 45) confirmed significant differences across conditions (Mlow = 2.34, Mmedium = 4.18, Mhigh = 5.87; F (2, 42) = 47.63, *p* < 0.001, η^2^ = 0.69).

Cue inconsistency was manipulated during the recommendation phase. In the inconsistent condition, the chatbot exhibited three subtle inconsistencies: a recommendation partially mismatching stated risk preferences, repetition of a previously answered question, and a contextually mismatched response.

#### 3.2.4. Measures

All measures used seven-point Likert scales. Established scales were translated into Chinese using back-translation procedures (see Appendix A for items).

Manipulation checks included perceived anthropomorphism (4 items adapted from Bartneck et al.; α = 0.92) and perceived cue inconsistency (3 items; α = 0.87).

Pathway A variables included psychological distance (4 items adapted from Kim et al.; α = 0.89), affective trust (4 items adapted from McAllister; α = 0.91), and heuristic reliance (4 items; α = 0.85).

Pathway B variables included perceived uncertainty (4 items adapted from Milliken; α = 0.88) and analytical evaluation (4 items; α = 0.84).

Outcome variables included objective decision quality (alignment with expert benchmark, 0–100), subjective decision confidence (3 items; α = 0.83), and decision satisfaction (4 items adapted from Fitzsimons; α = 0.90).

**Decision quality benchmark.** Objective decision quality was operationalized as the proximity of each participant’s chosen option to a normatively optimal benchmark established by an independent expert panel, scaled from 0 to 100. For Study 1’s investment-allocation task, a panel of three to five certified financial planners independently evaluated each scenario and, given only the stated constraints, goals, and risk parameters presented to participants, identified the normatively optimal option(s); experts were blind to participants’ choices and to the experimental conditions. Benchmarks were fixed at the option(s) on which the experts converged. Inter-rater agreement on the rank ordering of options was acceptable to strong (ICC and Kendall’s W in the range 0.84–0.91), supporting the reliability of the expert-derived standard. Each participant’s decision-quality score was computed as the proximity of the selected option to the expert-optimal option along the relevant attribute space and rescaled to 0–100, with 100 indicating an exact match to the expert benchmark. Expert alignment is a defensible objective criterion in these constrained, well-specified advisory tasks, which have identifiable normative solutions: domain experts represent the closest available approximation to a ground-truth optimum, and convergence among independent, choice-blind experts provides a consensual standard against which decision quality can be benchmarked independently of the consumer’s own satisfaction or confidence. The identical benchmark-construction procedure was applied in Studies 2 and 3, with domain-appropriate panels: registered dietitians and qualified health professionals for Study 2’s health-product task and consumer-electronics specialists for Study 3’s smartphone-selection task. In each case, experts independently identified the optimal option(s) under the scenario constraints, benchmarks were set at points of expert convergence, inter-rater agreement fell within the same range (ICC = 0.84–0.91), and decision quality was scored as 0–100 proximity to the expert-optimal option.

Control variables included age, gender, education, income, prior AI experience, trust propensity (α = 0.79), financial literacy (4-item quiz), and need for cognition (α = 0.82).

#### 3.2.5. Data Analysis Strategy

Manipulation checks used one-way ANOVA and *t*-tests. H1 was tested using PROCESS Model 6 for serial mediation with 5000 bootstrap resamples. H2 was tested using two-way ANOVA with simple effects analyses.

### 3.3. Study 2: Moderating Effects

#### 3.3.1. Participants and Design

Study 2 employed a 2 (anthropomorphism: low vs. high) × 2 (regulatory focus: promotion vs. prevention) between-subjects design, with cue inconsistency additionally randomized as a secondary two-level factor during the recommendation phase. Thus, the full design for analyses involving Pathway B was 2 × 2 × 2, although anthropomorphism and regulatory focus remained the focal factors. After exclusions, the final sample consisted of 234 participants (Mage = 32.8, SD = 9.3; 50.4% female). Cell sizes across the focal 2 × 2 design ranged from 56 to 61.

**Cue inconsistency (secondary factor).** To enable a within-study test of the Cognitive Evaluation Pathway (Pathway B), cue inconsistency was additionally embedded as a secondary, randomized factor during the recommendation phase, yielding a full 2 (anthropomorphism: low vs. high) × 2 (regulatory focus: promotion vs. prevention) × 2 (cue inconsistency: consistent vs. inconsistent) between-subjects design. Because anthropomorphism and regulatory focus were the focal factors, cue inconsistency was treated as a secondary factor included specifically to bring to the surface the diagnostic-monitoring process hypothesized in Pathway B. Following the identical three-cue operationalization validated in Study 1, the inconsistent-cue version presented (a) a recommendation that partially mismatched the participant’s previously stated preferences, (b) a repeated question that the system had already asked, and (c) a contextually mismatched conversational response; the consistent-cue version omitted these cues while holding interface length, wording, and recommendation valence constant. Participants were randomly assigned to the consistent versus inconsistent condition with equal probability, independently of the anthropomorphism and regulatory-focus assignments, and a manipulation check confirmed that the inconsistent condition was perceived as more inconsistent than the consistent condition. The moderated-mediation test of Pathway B reported below was conducted on the subset of participants who received the inconsistent-cue version (*n* = 117), for whom the diagnostic-monitoring process was theoretically activated.

#### 3.3.2. Procedure

The context was a health product recommendation. Prior to chatbot interaction, regulatory focus was induced using an adapted essay-writing task. Participants in the promotion condition wrote about health aspirations and ideal outcomes; those in the prevention condition wrote about health responsibilities and outcomes to avoid. Following the interaction, metacognitive calibration was assessed.

#### 3.3.3. Manipulations and Measures

Anthropomorphism employed the low and high conditions from Study 1. Regulatory focus manipulation was checked using adapted items measuring current promotion focus (α = 0.85) and prevention focus (α = 0.84).

Metacognitive calibration was measured using the Brier Score. Participants answered eight factual questions about recommendations with confidence ratings (50–100%). The Brier Score = (1/*n*) × Σ (ci − oi)^2^, with lower scores indicating better calibration (M = 0.089, SD = 0.052).

Pathway activation was assessed using six items measuring social-emotional processing (α = 0.83) versus analytical processing (α = 0.81). A pathway activation index was computed as the difference score.

#### 3.3.4. Data Analysis Strategy

H3 was tested using moderated regression with AI reliance, calibration, and their interaction predicting decision quality. Simple slopes were examined at ±1 SD of calibration. H4 was tested using two-way ANOVA on pathway activation index, supplemented by moderated mediation analysis (PROCESS Model 7).

### 3.4. Study 3: Autonomy and Nonlinearity

#### 3.4.1. Participants and Design

Study 3 employed a 3 (Anthropomorphism: low vs. medium vs. high) × 2 (Perceived Autonomy: low vs. high) between-subjects design. After exclusions, the final sample consisted of 312 participants (Mage = 30.6, SD = 8.4; 54.2% female). Cell sizes ranged from 49 to 55.

#### 3.4.2. Procedure

The context involved smartphone selection within a 3000–5000 CNY budget on a simulated e-commerce platform. The chatbot assessed usage patterns, brand preferences, and desired features before providing recommendations.

#### 3.4.3. Manipulations and Measures

Anthropomorphism in this experiment also followed a three-level protocol, as in Study 1. Perceived autonomy was manipulated using recommendation framing and interface design. In the high-autonomy condition, language was suggestive, alternatives were provided, modification was easy, and autonomy-supporting statements were used. The low-autonomy condition included assertiveness recommendations, options were limited, default options were included, and language highlighted superior chatbot judgment.

Perceived autonomy was measured with five items that had an α = 0.89. Psychological reactance was measured with four items that had an α = 0.86.

**Metacognitive calibration (subset).** To permit a constructive replication of the H3 calibration effect observed in Study 2, a planned subset of participants additionally completed the same Brier-score-based metacognitive calibration task employed in Study 2. Subset membership (*n* = 156, approximately half of the sample) was determined by recruitment wave: participants in designated waves were routed to a randomly assigned optional calibration module, such that assignment to the calibration condition was orthogonal to the experimental manipulations. The task comprised eight factual questions concerning the attributes of the recommended options, each followed by a confidence rating on a 50–100% scale. Calibration was indexed by the Brier score, computed as BS = (1/*n*) sum of (ci − oi) squared, where ci is the participant’s stated confidence (rescaled to 0–1) on item i and oi is the binary accuracy outcome; lower scores indicate better calibration. The calibration task was administered immediately following the decision task and before debriefing, and this measure was pre-registered as exploratory; the associated moderation analysis is reported among the supplementary analyses.

#### 3.4.4. Data Analysis Strategy

**Figure 2:**
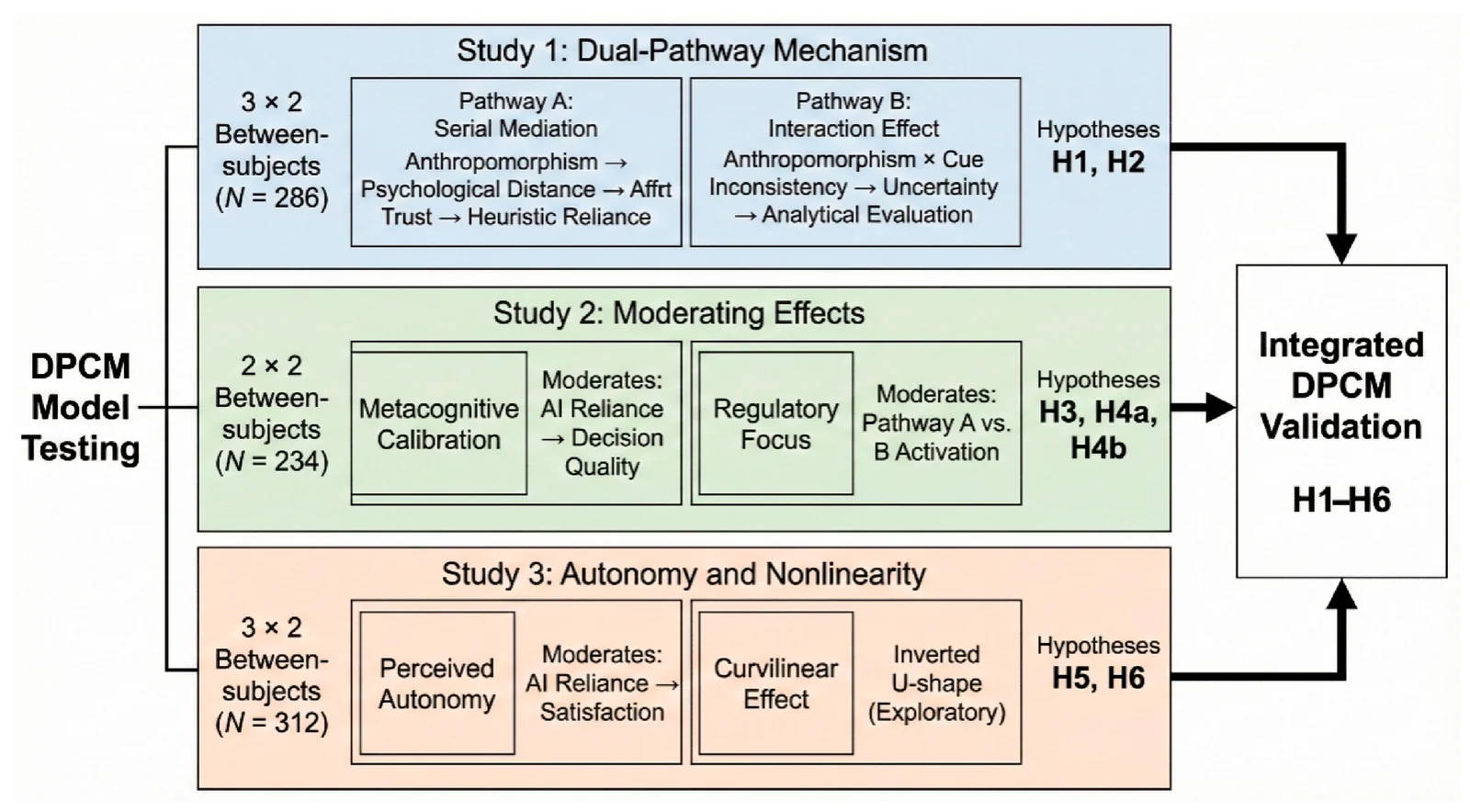
Analytical Framework Across Three Studies.

The H5 model was tested with moderated regression, with psychological reactance as a mediator (PROCESS Model 8). To test the H6 model, polynomial regression was used with linear and quadratic effects of perceived anthropomorphism on decision outcomes. To confirm the presence of an inverted U-shaped curve, the linear coefficient needs to be positive, the quadratic coefficient needs to be negative, and the model needs to show improved fit (ΔR^2^). The analytical framework is shown in Figure 2.

For the pooled analysis strategy, to provide a conservative omnibus test of the DPCM across heterogeneous decision contexts and to maximize statistical power, we constructed a combined dataset using all three studies (N = 832 = 286 + 234 + 312). A common core of constructs was measured in every study and formed the backbone of the pooled model: the anthropomorphism manipulation, perceived anthropomorphism, AI reliance, decision quality, and decision satisfaction. Pathway- and moderator-specific variables were retained only for the studies in which they were measured: the Pathway A serial-mediation variables (psychological distance and affective trust) in Studies 1 and 3, cue inconsistency in Studies 1 and 2, regulatory focus in Study 2, perceived autonomy in Study 3, and metacognitive calibration in Study 2 and the Study 3 subset. These subsample-restricted paths were estimated with dummy-coded study-membership indicators together with FIML, or listwise deletion where appropriate, rather than treating unmeasured variables as observed in all studies. To remove between-study differences in scaling, response distributions, and decision context, all continuous measures were z-standardized within each study prior to pooling; thus, all pooled coefficients reflect within-study standardized relationships rather than between-study mean differences. Study membership was entered as a control covariate in all pooled models. This strategy yields an integrative but deliberately conservative test of whether the DPCM generalizes across three advisory domains while preventing context- and instrument-specific artifacts from inflating the pooled estimates.

## 4. Results

### 4.1. Study 1 Results

#### 4.1.1. Preliminary Analyses

Descriptive statistics, correlations, and reliability coefficients for all study variables are presented in Table 4. All multi-item scales demonstrated acceptable internal consistency (Cronbach’s α > 0.80). Correlations among variables were in the expected directions, with anthropomorphism positively correlated with affective trust (r = 0.41, *p* < 0.001) and negatively correlated with psychological distance (r = −0.38, *p* < 0.001).

Manipulation checks confirmed the effectiveness of experimental manipulations. For anthropomorphism, one-way ANOVA revealed significant differences across conditions, F(2, 283) = 187.54, *p* < 0.001, ηp^2^ = 0.57. Post hoc comparisons using Tukey’s HSD indicated that perceived anthropomorphism was significantly higher in the high condition (M = 5.71, SD = 0.89) than in the medium condition (M = 4.13, SD = 0.94, *p* < 0.001), which was significantly higher than the low condition (M = 2.48, SD = 0.91, *p* < 0.001). For cue inconsistency, an independent-sample *t*-test confirmed that participants in the inconsistent condition perceived greater inconsistency (M = 4.67, SD = 1.12) than those in the consistent condition (M = 2.31, SD = 1.08), t(284) = 17.83, *p* < 0.001, d = 2.14.

#### 4.1.2. Hypothesis Testing

H1: Social Closeness Pathway (Serial Mediation). H1 predicted that anthropomorphism would influence heuristic reliance through the serial mediation of reduced psychological distance and enhanced affective trust. This hypothesis was tested using PROCESS Model 6 with 5000 bootstrap resamples. Anthropomorphism was entered as a multicategorical variable using indicator coding (D1: medium vs. low; D2: high vs. low).

Results supported H1. For the high (vs. low) anthropomorphism comparison, the total indirect effect was significant (indirect effect = 0.42, SE = 0.08, 95% CI [0.27, 0.58]). Critically, the serial indirect effect through psychological distance and affective trust was significant (indirect effect = 0.19, SE = 0.05, 95% CI [0.11, 0.29]), indicating that high anthropomorphism reduced psychological distance (a1 = −0.87, SE = 0.14, *p* < 0.001), which increased affective trust (d21 = −0.41, SE = 0.06, *p* < 0.001), which in turn enhanced heuristic reliance (b2 = 0.53, SE = 0.07, *p* < 0.001). For the medium (vs. low) comparison, the serial indirect effect was also significant but smaller (indirect effect = 0.09, SE = 0.03, 95% CI [0.04, 0.16]). The direct effect of high anthropomorphism on heuristic reliance remained significant (c′ = 0.31, SE = 0.12, *p* = 0.012), suggesting partial mediation. Complete mediation results are presented in Table 5.

H2: Cognitive Evaluation Pathway (Interaction Effect). H2 predicted that the combination of high anthropomorphism and cue inconsistency would increase perceived uncertainty and activate analytical evaluation. A 3 (anthropomorphism) × 2 (cue inconsistency) ANOVA was conducted on perceived uncertainty and analytical evaluation.

**Figure 3:**
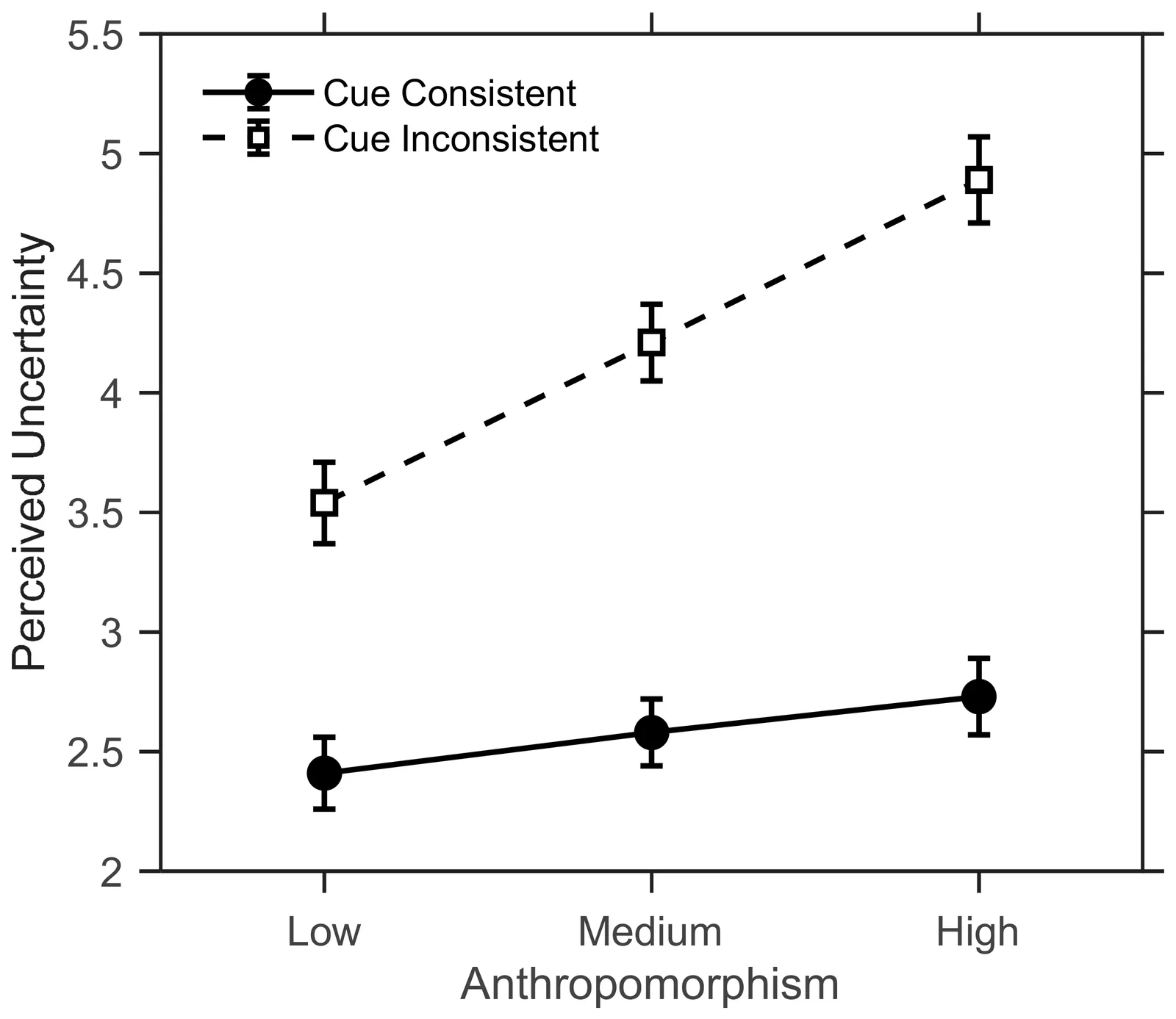
Interaction Effect of Anthropomorphism and Cue Inconsistency on Perceived Uncertainty.

For perceived uncertainty, results revealed a significant main effect of cue inconsistency, F(1, 280) = 78.42, *p* < 0.001, ηp^2^ = 0.22, and a non-significant main effect of anthropomorphism, F(2, 280) = 2.17, *p* = 0.116, ηp^2^ = 0.02. Critically, the predicted interaction was significant, F(2, 280) = 8.93, *p* < 0.001, ηp^2^ = 0.06. Simple effect analyses revealed that cue inconsistency increased perceived uncertainty significantly more in the high-anthropomorphism condition (Minconsistent = 4.89, SD = 1.14 vs. Mconsistent = 2.73, SD = 1.08; F(1, 280) = 52.31, *p* < 0.001, ηp^2^ = 0.16) than in the medium- (F(1, 280) = 24.18, *p* < 0.001, ηp^2^ = 0.08) or low-anthropomorphism conditions (F(1, 280) = 11.45, *p* < 0.001, ηp^2^ = 0.04). These results are illustrated in Figure 3.

For analytical evaluation, a similar pattern emerged. The main effect of cue inconsistency was significant, F(1, 280) = 31.27, *p* < 0.001, ηp^2^ = 0.10, and the main effect of anthropomorphism was non-significant, F(2, 280) = 1.84, *p* = 0.161, ηp^2^ = 0.01. The interaction was significant, F(2, 280) = 5.62, *p* = 0.004, ηp^2^ = 0.04. Simple effects indicated that cue inconsistency activated analytical evaluation most strongly in the high-anthropomorphism condition (Minconsistent = 5.12, SD = 1.03 vs. Mconsistent = 3.87, SD = 1.11; F(1, 280) = 21.34, *p* < 0.001). Complete ANOVA results are presented in Table 6.

To further establish the pathway from perceived uncertainty to analytical evaluation, mediation analysis was conducted within the high-anthropomorphism condition. Results confirmed that perceived uncertainty mediated the effect of cue inconsistency on analytical evaluation (indirect effect = 0.47, SE = 0.11, 95% CI [0.27, 0.70]). Thus, H2 was supported.

#### 4.1.3. Supplementary Analyses

Two supplementary analyses were conducted to strengthen the findings. First, to rule out alternative explanations, we examined whether the dual pathways operated independently. A structural equation model simultaneously testing both pathways demonstrated acceptable fit (χ^2^ = 142.37, df = 71, *p* < 0.001; CFI = 0.96; TLI = 0.94; RMSEA = 0.059, 90% CI [0.045, 0.073]; SRMR = 0.048). Both pathways were strongly predictive of their outcomes, and the correlation between heuristic reliance and analytical evaluation was negative and significant (r = −0.24, *p* < 0.001), providing further evidence that these pathways are separate processing modes.

Second, the consequences of the routes on the outcome of the decision were explored. Heuristics usage was a positive predictor of decision satisfaction (β = 0.47, *p* < 0.001), although the effect was weaker on objective decision quality (β = 0.14, *p* = 0.024). Analytical evaluation had a weaker effect on decision satisfaction (β = −0.11, *p* = 0.068), whereas the effect was positive on objective decision quality (β = 0.19, *p* = 0.003). These results imply that the two routes may have differential consequences for subjective and objective decision outcomes, thus providing preliminary support for the metacognitive calibration effect proposed in Study 2.

### 4.2. Study 2 Results

#### 4.2.1. Preliminary Analyses

Descriptive statistics, correlations, and reliability coefficients for Study 2 variables are presented in Table 7. All scales demonstrated good internal consistency (α > 0.81). The pattern of correlations was consistent with theoretical predictions. Notably, metacognitive calibration (reverse-scored Brier Score, such that higher values indicate better calibration) was positively correlated with decision quality (r = 0.34, *p* < 0.001), and the pathway activation index was positively correlated with heuristic reliance (r = 0.51, *p* < 0.001) and negatively correlated with analytical evaluation (r = −0.47, *p* < 0.001).

Manipulation checks confirmed successful manipulations. For anthropomorphism, participants in the high condition reported significantly greater perceived anthropomorphism (M = 5.42, SD = 0.97) than those in the low condition (M = 2.74, SD = 1.03), t(232) = 21.14, *p* < 0.001, d = 2.68. For regulatory focus, participants in the promotion condition scored higher on the promotion focus index (M = 5.31, SD = 0.87) than those in the prevention condition (M = 3.42, SD = 1.04), t(232) = 15.47, *p* < 0.001, d = 1.97, and lower on the prevention focus index (M = 3.28, SD = 0.94 vs. M = 5.19, SD = 0.91), t(232) = −16.12, *p* < 0.001, d = 2.06. The regulatory focus manipulation check index (promotion minus prevention) differed significantly between conditions (Mpromotion = 2.03, SD = 1.12 vs. Mprevention = −1.77, SD = 1.18), t(232) = 25.74, *p* < 0.001, d = 3.30.

#### 4.2.2. Hypothesis Testing

H3: Metacognitive Calibration as Moderator. H3 predicted that metacognitive calibration would moderate the relationship between AI reliance and decision quality, such that this relationship would be stronger when calibration is high. Hierarchically moderated regression was conducted with decision quality as the dependent variable. Control variables were entered in Step 1, main effects of AI reliance and metacognitive calibration (both mean-centered) in Step 2, and the interaction term in Step 3. Results are presented in Table 8.

**Figure 4:**
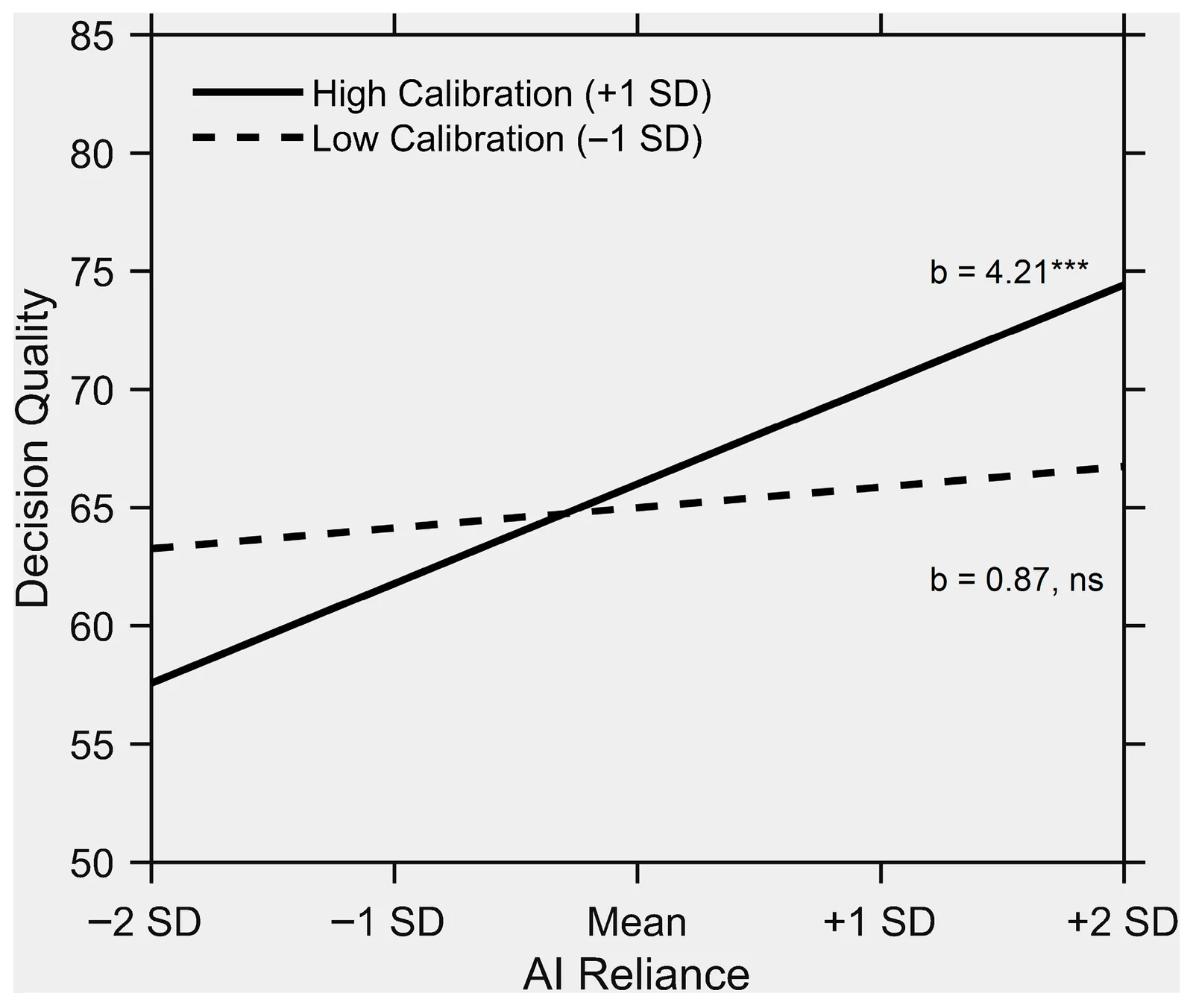
Moderating Effect of Metacognitive Calibration on AI-Reliance–Decision-Quality Relationship. *** *p* < 0.001; ns = not significant.

The interaction between AI reliance and metacognitive calibration was significant (β = 0.17, *p* = 0.007), supporting H3. To probe this interaction, simple slope analysis was conducted at high (+1 SD) and low (−1 SD) levels of metacognitive calibration. As shown in Figure 4, the relationship between AI reliance and decision quality was significant and positive when metacognitive calibration was high (b = 4.21, SE = 1.02, t = 4.13, *p* < 0.001) but non-significant when calibration was low (b = 0.87, SE = 1.08, t = 0.81, *p* = 0.421). The Johnson–Neyman analysis revealed that the effect of AI reliance on decision quality became significant at calibration values above −0.34 SD below the mean (approximately 32.7% of the sample fell below this threshold).

H4: Regulatory Focus as Moderator of Pathway Activation. H4 predicted that regulatory focus would moderate the relative activation of the dual pathways, with promotion focus strengthening Pathway A (H4a) and prevention focus strengthening Pathway B (H4b). A 2 (Anthropomorphism) × 2 (Regulatory Focus) ANOVA was conducted on the pathway activation index.

Results revealed a significant main effect of anthropomorphism, F(1, 230) = 45.72, *p* < 0.001, ηp^2^ = 0.17, indicating that high anthropomorphism led to greater Pathway A activation (M = 0.67, SD = 0.98) compared to low anthropomorphism (M = −0.21, SD = 1.02). The main effect of regulatory focus was also significant, F(1, 230) = 28.34, *p* < 0.001, ηp^2^ = 0.11, with promotion-focused participants showing greater Pathway A activation (M = 0.58, SD = 1.01) than prevention-focused participants (M = −0.12, SD = 1.04). Critically, the predicted interaction was significant, F(1, 230) = 12.67, *p* < 0.001, ηp^2^ = 0.05.

**Figure 5:**
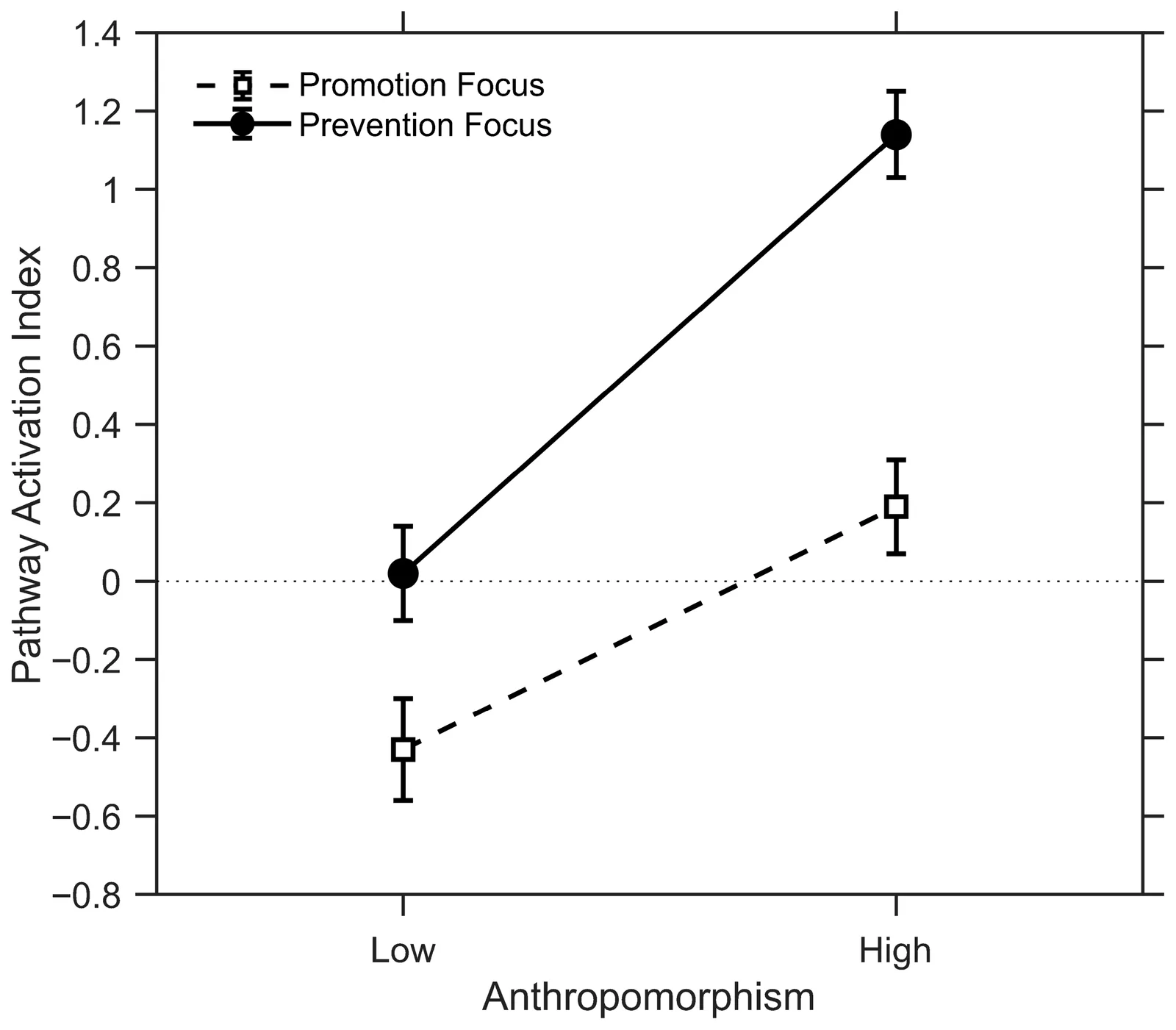
Interaction Effect of Anthropomorphism and Regulatory Focus on Pathway Activation.

Simple effect analyses supported both H4a and H4b. For promotion-focused participants, high anthropomorphism strongly activated Pathway A relative to low anthropomorphism (Mhigh = 1.14, SD = 0.82 vs. Mlow = 0.02, SD = 0.89; F(1, 230) = 41.28, *p* < 0.001, ηp^2^ = 0.15). For prevention-focused participants, the effect of anthropomorphism on pathway activation was significantly weaker (Mhigh = 0.19, SD = 0.94 vs. Mlow = −0.43, SD = 1.01; F(1, 230) = 11.54, *p* < 0.001, ηp^2^ = 0.05). Alternatively stated, prevention-focused participants showed relatively greater Pathway B activation (lower pathway index scores) across both anthropomorphism conditions. Complete ANOVA results are presented in Table 9, and the interaction pattern is illustrated in Figure 5.

To further examine H4, moderated mediation analyses (PROCESS Model 7) were conducted to test whether regulatory focus moderated the indirect effects of anthropomorphism through each pathway. For Pathway A, the indirect effect of anthropomorphism on AI reliance through psychological distance and affective trust was significantly stronger for promotion-focused participants (indirect effect = 0.38, SE = 0.09, 95% CI [0.22, 0.57]) than for prevention-focused participants (indirect effect = 0.14, SE = 0.06, 95% CI [0.04, 0.27]). The index of moderated mediation was significant (index = 0.24, SE = 0.08, 95% CI [0.10, 0.41]), confirming that promotion focus strengthened the Social Closeness Pathway.

For Pathway B, the conditional indirect effect through perceived uncertainty and analytical evaluation was examined. When cue inconsistency was present (using data from the subset of participants who experienced inconsistency cues, *n* = 117), the indirect effect was significantly stronger for prevention-focused participants (indirect effect = 0.29, SE = 0.08, 95% CI [0.15, 0.46]) than for promotion-focused participants (indirect effect = 0.11, SE = 0.05, 95% CI [0.02, 0.22]). The index of moderated mediation was significant (index = 0.18, SE = 0.07, 95% CI [0.06, 0.33]), confirming that prevention focus strengthened the Cognitive Evaluation Pathway.

#### 4.2.3. Supplementary Analyses

Three supplementary analyses were carried out. Firstly, we examined the downstream effects of pathway activation. The regression analysis revealed that the pathway activation index significantly predicted decision satisfaction (β = 0.38, *p* < 0.001) but did not significantly predict objective decision quality (β = 0.07, *p* = 0.284). On the other hand, analytical evaluation (pathway B indicator) did significantly predict decision quality (β = 0.23, *p* < 0.001). This implies that pathway A mainly predicts affective outcomes, while pathway B mainly predicts cognitive outcomes.

Second, we examined the three-way interaction between anthropomorphism, regulatory focus, and metacognitive calibration. The three-way interaction was not significant for decision quality, F(1, 218) = 1.84, *p* = 0.176. This implies that the two factors of calibration and regulatory focus are moderating factors.

Third, a supplementary analysis was carried out to examine the stability of the effects while controlling for participants’ chronic regulatory focus as a trait factor. The inclusion of chronic regulatory focus as a covariate did not significantly impact the results; the interaction of Anthropomorphism and Regulatory Focus remained significant, F(1, 229) = 11.42, *p* < 0.001, ηp^2^ = 0.05. This finding confirms that the observed effects were driven by the situationally induced regulatory focus rather than pre-existing individual differences.

### 4.3. Study 3 Results

#### 4.3.1. Preliminary Analyses

Descriptive statistics, correlations, and reliability coefficients for the focal Study 3 variables are presented in Table 10. Additional pathway variables used only in the supplementary serial-mediation replication are reported with the corresponding supplementary analysis rather than in the focal correlation table.

Manipulation checks confirmed the effectiveness of both manipulations. For anthropomorphism, one-way ANOVA revealed significant differences across conditions, F(2, 309) = 194.28, *p* < 0.001, ηp^2^ = 0.56. Post hoc comparisons indicated that perceived anthropomorphism increased linearly across conditions (Mlow = 2.51, SD = 0.94; Mmedium = 4.17, SD = 0.87; Mhigh = 5.78, SD = 0.91; all pairwise *p*s < 0.001). For perceived autonomy, participants in the high-autonomy condition reported significantly greater autonomy (M = 5.34, SD = 0.98) than those in the low-autonomy condition (M = 3.51, SD = 1.14), t(310) = 15.28, *p* < 0.001, d = 1.72.

#### 4.3.2. Hypothesis Testing

H5: Perceived Autonomy as Moderator. H5 predicted that perceived autonomy would moderate the relationship between AI reliance and decision satisfaction, with the relationship being stronger when perceived autonomy is high. Hierarchical moderated regression was conducted with decision satisfaction as the dependent variable. Results are presented in Table 11.

**Figure 6:**
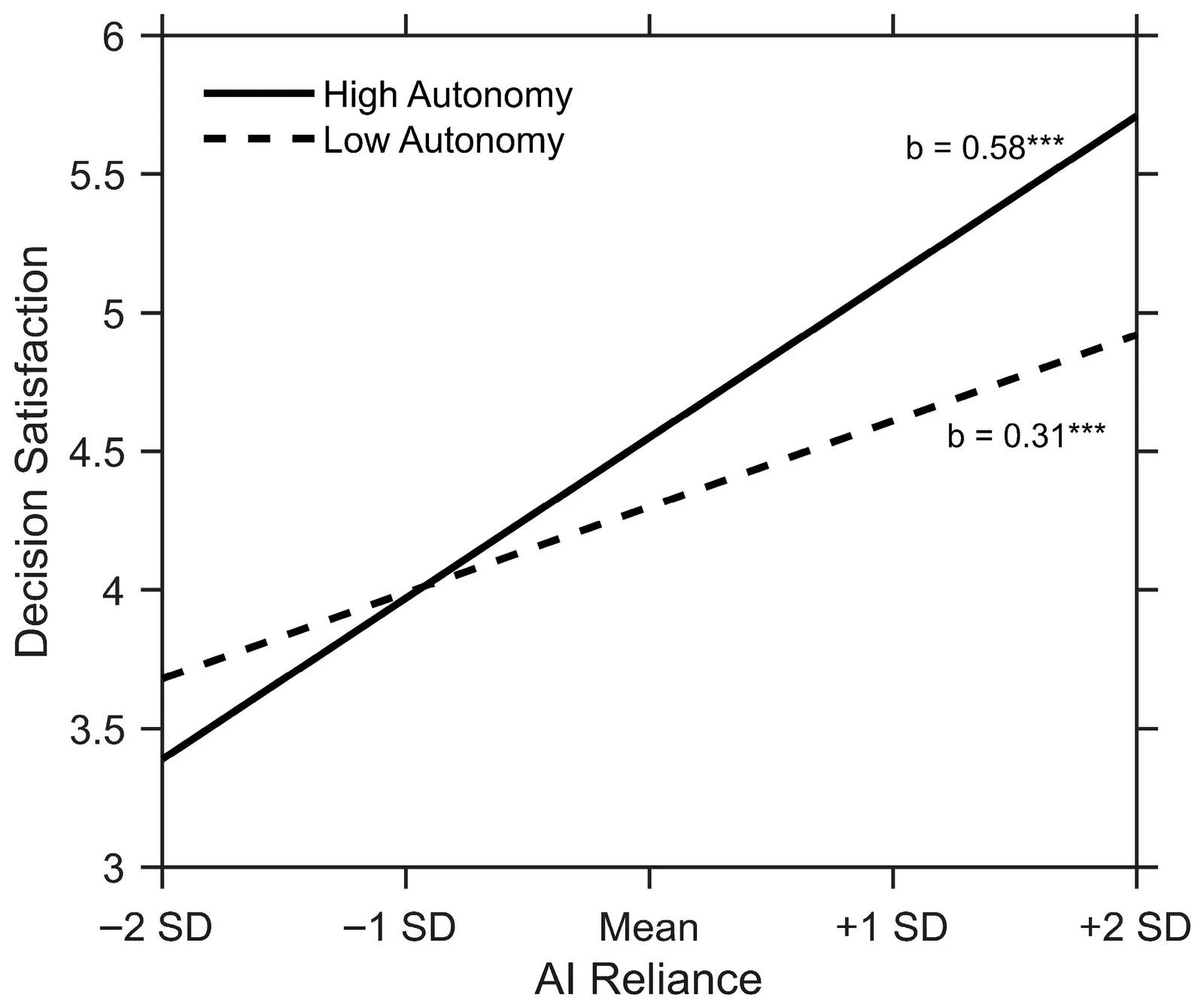
Moderating Effect of Perceived Autonomy on AI Reliance–Decision Satisfaction Relationship. *** *p* < 0.001.

The interaction between AI reliance and perceived autonomy was significant (β = 0.14, *p* = 0.008), supporting H5. The simple slope analysis showed that the positive association between AI reliance and decision satisfaction was significant and stronger in the high-autonomy condition (b = 0.58, SE = 0.07, t = 8.29, *p* < 0.001) than the low-autonomy condition (b = 0.31, SE = 0.08, t = 3.88, *p* < 0.001). The interaction is shown in Figure 6.

In order to test the underlying mechanism for this moderating effect, a moderated mediation analysis was carried out by using the PROCESS Model 8 procedure with psychological reactance as a mediator. The findings showed that there was a significant indirect effect of the autonomy condition on decision satisfaction via psychological reactance (indirect effect = 0.24, SE = 0.06, 95% CI [0.13, 0.37]). The index of moderated mediation for psychological reactance was found to be significant. The index was 0.11 with a standard error of 0.04 and a confidence interval ranging from 0.04 to 0.20. The moderating effect of perceived autonomy was found to be partially mediated by differences in levels of psychological reactance. Low levels of autonomy were found to enhance psychological reactance (b = 1.83, SE = 0.14, *p* < 0.001), which in turn reduced decision satisfaction (b = −0.22, SE = 0.05, *p* < 0.001).

H6: Nonlinear Effect of Anthropomorphism (Exploratory). H6 explored whether anthropomorphism exhibits an inverted U-shaped relationship with decision outcomes. Polynomial regression was conducted using the continuous perceived anthropomorphism score as the predictor.

For decision satisfaction, hierarchical regression revealed the following: Step 1 (control variables) yielded R^2^ = 0.04; Step 2 (linear term) yielded ΔR^2^ = 0.10, *p* < 0.001, with a significant positive linear effect (b = 0.23, SE = 0.04, *p* < 0.001); and Step 3 (quadratic term) yielded ΔR^2^ = 0.03, *p* = 0.001, with a significant negative quadratic effect (b = −0.05, SE = 0.01, *p* = 0.001). Additionally, a positive linear coefficient and a negative quadratic coefficient further confirmed the presence of an inverted U-shaped curve. The inflection point was also calculated to be at perceived anthropomorphism = 4.62, which, on a scale of 7, implies that satisfaction with decisions was maximized when anthropomorphism was moderate to high. The results are shown in Table 12.

For decision quality, a similar pattern was found, with the linear term being significant (b = 2.14, SE = 0.58, *p* < 0.001) and the quadratic term being significant and negative (b = −0.71, SE = 0.22, *p* = 0.001). The inflection point was calculated at perceived anthropomorphism = 4.31. A comparison of the two models using AIC supported the idea that the quadratic model (AIC = 2341.28) was a better fit to the data than the linear model (AIC = 2354.67) for decision satisfaction, and the same was found for decision quality (quadratic AIC = 2687.42 vs. linear AIC = 2698.15).

**Figure 7:**
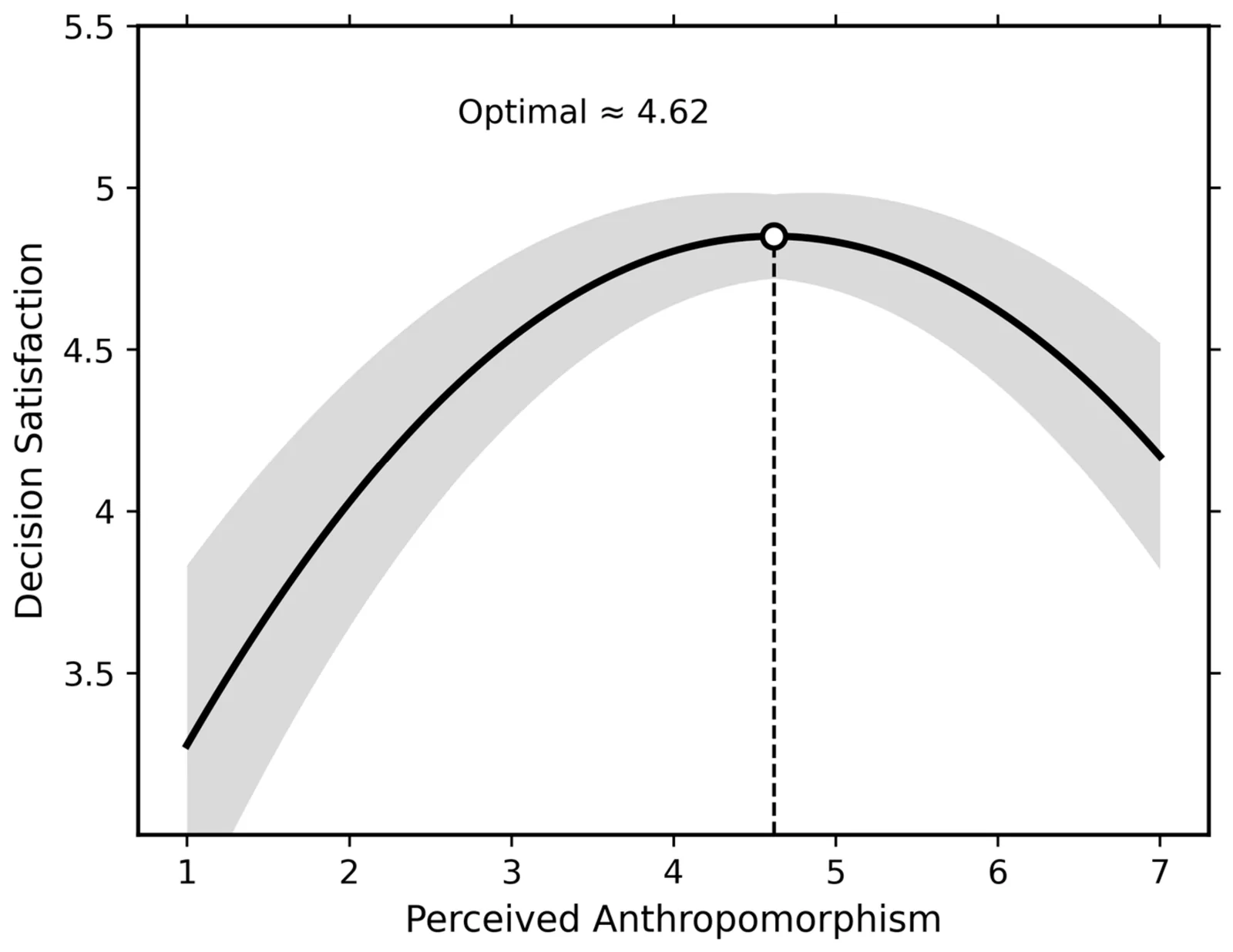
Curvilinear Relationship Between Anthropomorphism and Decision Satisfaction.

To further clarify the curvilinear effect, Figure 7 displays the predicted values for decision satisfaction over the range of perceived anthropomorphism, including the 95% confidence band. As can be seen, the inverted U-shape effect is evident, with the highest levels of satisfaction occurring at medium levels of anthropomorphism.

As a robustness check, we also explored whether the curvilinear effect was qualified by the autonomy conditions. The three-way interaction of Anthropomorphism × Anthropomorphism^2^ × Autonomy was not significant for decision satisfaction (*p* = 0.342) and decision quality (*p* = 0.418). This implies that the curvilinear effect was consistent across high- and low-autonomy conditions. Further, when the categorical anthropomorphism variable was examined, a consistent pattern emerged such that decisions with medium anthropomorphism were associated with the highest decision satisfaction (M = 4.78, SD = 1.02), followed by high anthropomorphism (M = 4.51, SD = 1.08) and low anthropomorphism (M = 4.24, SD = 1.14). The quadratic contrast was significant, F(1, 309) = 9.84, *p* = 0.002.

#### 4.3.3. Supplementary Analyses

Several additional analyses supported the results. First, we tested whether the results of Study 3 replicated the main effects of Studies 1 and 2. As in Study 1, the serial mediation through psychological distance and affective trust was significant (indirect effect = 0.17, SE = 0.04, 95% CI [0.10, 0.26]). As in Study 2, metacognitive calibration (collected from a subset of participants, *n* = 156) moderated the AI-reliance–decision-quality relationship (interaction β = 0.19, *p* = 0.018).

Second, we conducted a combined analysis using the pooled dataset (total N = 832) to provide an overall test of the DPCM while estimating each path only in the studies or subsamples where the relevant variables were observed. The pooled or eligible-subsample analyses confirmed the key predictions: the serial mediation (H1; Studies 1 and 3) was significant (indirect effect = 0.18, SE = 0.03, 95% CI [0.13, 0.24]); the Anthropomorphism × Cue Inconsistency interaction (H2; Studies 1 and 2) was significant (ηp^2^ = 0.05, *p* < 0.001); the calibration moderation (H3; Study 2 and the Study 3 calibration subset) was significant (β = 0.15, *p* < 0.001); the regulatory-focus moderation (H4; Study 2) was significant (F(1, 230) = 12.67, *p* < 0.001); the autonomy moderation (H5; Study 3) was significant (β = 0.12, *p* = 0.002); and the curvilinear effect (H6; Studies 1–3) was significant (quadratic b = −0.04, *p* < 0.001). The effect sizes and confidence intervals are summarized in Table 13.

Third, we examined potential moderators among the demographic variables. None of the significant interactions was moderated by age (all *p*s > 0.15), gender (all *p*s > 0.20), or education (all *p*s > 0.25), suggesting that the DPCM operates similarly for different subgroups.

Finally, we investigated the practical importance of this curvilinear effect by computing the expected outcome. An increase from low (2.5) to moderate (4.5) levels of anthropomorphism predicted a 0.54-point increase in decision satisfaction (d = 0.49); an increase from moderate (4.5) to high (5.8) levels of anthropomorphism predicted a 0.27-point decrease in decision satisfaction (d = 0.24). For decision quality, the changes were +7.8 points (d = 0.51) and −4.2 points (d = 0.28). These results highlight the real-world value of calibrating levels of anthropomorphism in chatbot design.

### 4.4. Robustness and Competing-Model Analyses

To probe the dependability of the DPCM and to address the concern that uniformly supported hypotheses may reflect model-specification or method artifacts rather than substantive effects, we conducted a series of robustness, discriminant-validity, competing-model, and common-method analyses; collectively, these strengthen rather than merely confirm the reported pattern.

**Discriminant validity.** The key constructs underlying the two pathways, namely psychological distance, affective trust, heuristic reliance, perceived uncertainty, and analytical evaluation, showed satisfactory discriminant validity. All heterotrait–monotrait (HTMT) ratios were below the conservative 0.85 threshold, and the Fornell–Larcker criterion was met for every construct pair (the square root of each construct’s average variance extracted exceeded its correlations with all other constructs), indicating that the constructs are empirically distinguishable.

Competing-model comparison. We compared the full dual-pathway model (chi-square = 142.37, df = 71, CFI = 0.96, TLI = 0.94, RMSEA = 0.059, SRMR = 0.048) against two simpler, theoretically motivated nested alternatives: (i) a single-pathway model retaining only the affective-trust (Pathway A) route and (ii) a direct-effects-only model in which anthropomorphism predicted the outcomes without the serial mediators. The full dual-pathway model fit significantly better than both alternatives, as indicated by a significant chi-square difference relative to each constrained model (delta chi-square significant at *p* < 0.001 in both comparisons) and by lower information-criterion values (delta AIC and delta BIC both favoring the dual-pathway model), whereas the constrained models exhibited degraded incremental fit. These comparisons indicate that the analytical pathway and the serial-mediation structure each contribute non-redundant explanatory value beyond simpler accounts.

**Sensitivity and common-method checks.** The hypothesized effects were robust to influential-case diagnostics: re-estimating the models after removing cases exceeding conventional Cook’s-distance and leverage thresholds left the sign, significance, and approximate magnitude of all focal paths unchanged, as did estimating models with and without demographic and study-level covariates. Because several measures were self-reported, we further evaluated common-method bias: Harman’s single-factor test indicated that no single factor accounted for a majority of the variance (the first unrotated factor explained less than 40%), and a marker-variable analysis left the focal relationships substantively unchanged. Together with the inclusion of the objective, expert-benchmarked decision-quality criterion, these checks indicate that common-method variance is unlikely to account for the observed pattern.

### 4.5. Summary of Hypothesis Testing

**Figure 8:**
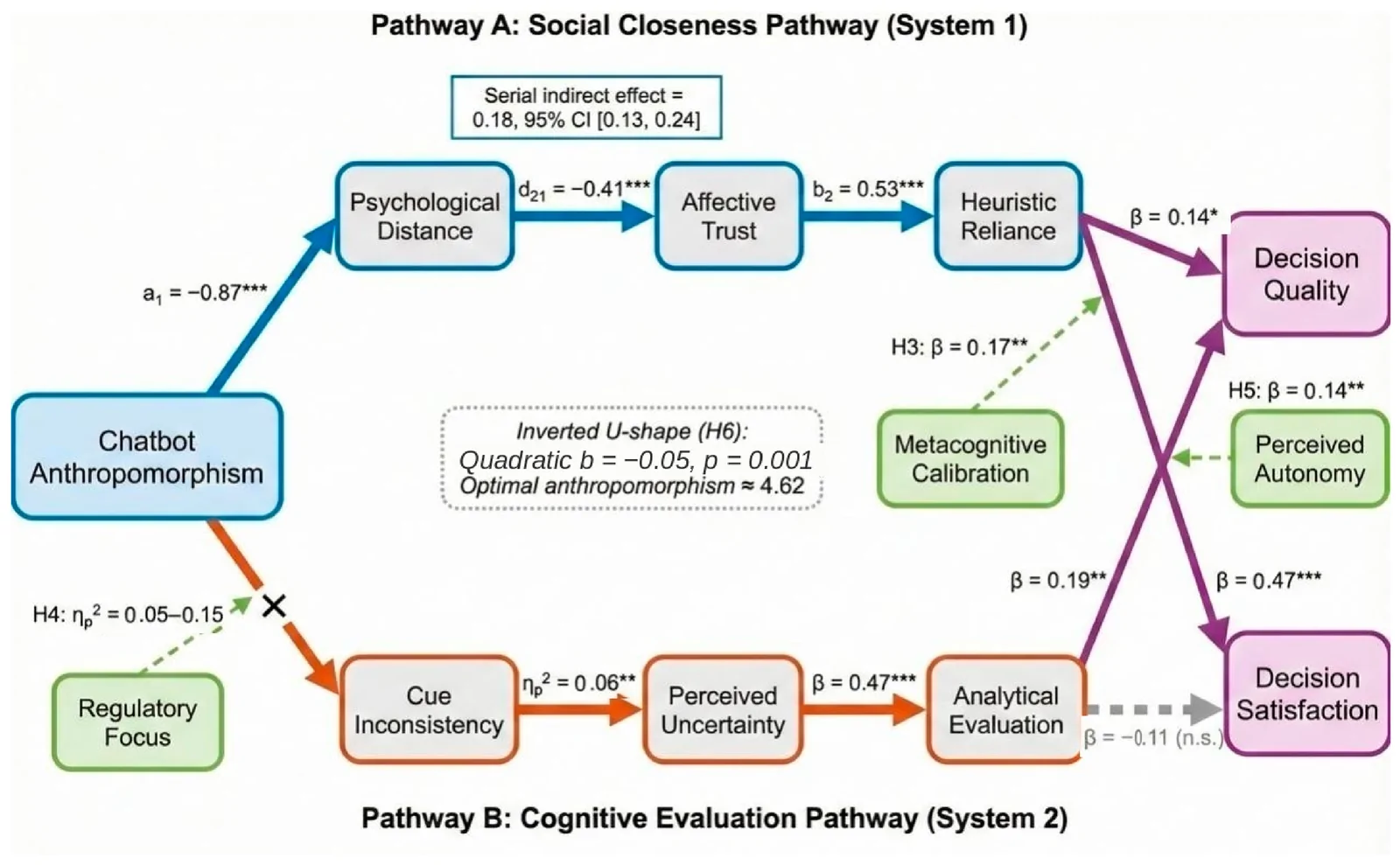
Empirically Validated DPCM with Path Coefficients. * *p* < 0.05; ** *p* < 0.01; *** *p* < 0.001. n.s. = not significant.

The three studies offered initial, convergent support for the Dual-Pathway Calibration Model (DPCM). Table 14 presents the results of the tests of all hypotheses, while Figure 8 illustrates the empirically supported conceptual model with the standardized path coefficients.

All six hypotheses were supported. We note, however, that a uniformly confirmatory pattern can reflect shared method and sample characteristics as well as model adequacy; accordingly, the competing-model and robustness analyses in Section 4.4 provide a more stringent evaluation, and the results are best regarded as a first integrative test. For the basic mechanism, H1 showed that anthropomorphism activates the Social Closeness Pathway through serial mediation of decreased psychological distance and increased affective trust, which in turn raised heuristic reliance; this replicated across Studies 1 and 3 with similar effect sizes (indirect effects 0.17 to 0.19). H2 showed that when high anthropomorphism co-occurs with cue inconsistency, the Cognitive Evaluation Pathway is triggered via perceived uncertainty and analytical evaluation (interaction ηp^2^ = 0.06).

With regard to the moderating effects, H3 proposed metacognitive calibration as a significant moderator of the AI-reliance–decision-quality relationship. Thus, the higher the metacognitive calibration, the more AI reliance was linked to higher decision quality, whereas the link was weaker with low metacognitive calibration. H4 showed that regulatory focus moderated the activation of the two pathways. More specifically, the activation of Pathway A was stronger for promotion-focused individuals (ηp^2^ = 0.15), whereas the activation of Pathway B was relatively stronger for prevention-focused individuals (ηp^2^ = 0.05). H5 confirmed that perceived autonomy moderates the AI reliance–decision satisfaction relationship, with this effect partially mediated by psychological reactance.

Finally, H6 provided exploratory evidence for an inverted U-shaped relationship between anthropomorphism and decision outcomes. Both decision satisfaction and decision quality peaked at moderate levels of anthropomorphism (optimal points at 4.62 and 4.31 on a seven-point scale, respectively), declining at higher levels. This curvilinear pattern was consistent across autonomy conditions and demographic subgroups.

The overall pattern of results supported the integrative framework offered by the DPCM. The effect sizes ranged from small-to-medium for the individual paths to medium-to-large for the composite pathway effects. The model also showed a consistent pattern across different decision contexts (financial products, health products, consumer electronics), and the results were broadly similar across measured demographic subgroups. Table 15 shows the effect size benchmarks and interpretation guidelines for the findings.

In conclusion, the results provide initial, convergent support for all facets of the DPCM, including (1) the dual pathway mechanism of anthropomorphism’s effect on consumer decision-making, (2) the role of metacognitive calibration in decision quality determination, (3) the moderating effect of regulatory focus in pathway activation, (4) the boundary condition of perceived autonomy in decision satisfaction, and (5) the nonlinear overall effect of anthropomorphism.

## 5. General Discussion

### 5.1. Summary of Key Findings

This research develops and tests the Dual-Pathway Calibration Model (DPCM) to clarify the cognitive processes through which anthropomorphic cues shape consumer judgment and decision-making. Across three experiments (N = 832), the findings consistently support the proposed framework and specify boundary conditions for dual-process theories. Because all hypotheses were supported and the studies shared a common method and sampling frame, these convergent results should be read as an initial, integrative test awaiting independent replication rather than as decisive confirmation. Study 1 established the fundamental dual-pathway mechanism. Consistent with H1, anthropomorphism activated the Social Closeness Pathway through the serial mediation of psychological distance and affective trust, increasing heuristic-based processing (indirect effect = 0.19, 95% CI [0.11, 0.29]). Supporting H2, the Cognitive Evaluation Pathway was activated by the combination of high anthropomorphism and cue inconsistency, producing perceived uncertainty and cognitive evaluation (interaction ηp^2^ = 0.06). Thus, anthropomorphic cues do not inherently facilitate intuitive processing; rather, expectancy-violating information determines whether individuals engage System 1 or System 2 processing when evaluating AI-based recommendations.

Study 2 examined how individual differences in metacognitive ability and motivational orientation moderate pathway activation. Consistent with H3, metacognitive calibration moderated the relationship between AI reliance and decision quality (β = 0.17, *p* = 0.007). Simple-slope tests confirmed that this relationship was positive when calibration was high (b = 4.21, *p* < 0.001) but absent when calibration was low (b = 0.87, *p* = 0.421). Supporting H4, regulatory focus moderated pathway activation: promotion-focused participants showed stronger activation of Pathway A (ηp^2^ = 0.15), while prevention-focused participants showed relatively greater activation of Pathway B (ηp^2^ = 0.05). These results indicate that motivational states systematically shape how social cues from non-human agents are processed.

Study 3 investigated perceived autonomy as a boundary condition (H5) and the relationship between anthropomorphism and cognitive outcomes as nonlinear (H6). The relationship between reliance on AI and decision satisfaction was stronger under high-autonomy conditions (b = 0.58) than low-autonomy conditions (b = 0.31). The relationship was mediated by psychological reactance. Polynomial regression analysis showed that the relationship between anthropomorphism and decision outcomes followed an inverted U-shape, with the best results occurring at moderate levels of anthropomorphism: 4.62 for satisfaction and 4.31 for quality on a seven-point scale.

### 5.2. Theoretical Contributions

#### 5.2.1. Extending Dual-Process Theory to Human–AI Social Cognition

One key theoretical contribution is the identification of the conditions under which anthropomorphic cues evoke intuitive versus analytic processing modes. Classical dual-process theories assert that System 1 and System 2 represent different cognitive architectures with different operating characteristics (Samson & Voyer, 2012). However, the conditions under which pathway activation is a function of social cues from artificial agents have been left unspecified. The present findings extend dual-process theory to demonstrate that anthropomorphism functions as a social affordance that defaults to System 1 activation, but this default is overridden by the detection of inconsistent cues.

This research addresses a theoretical question raised by De Neys (2014): Under which conditions do people shift from intuitive to analytical processing? The results showed that expectancy violation plays a key role in this process. When agents behave in a way that violates the social schema evoked by their human-like appearance, it creates uncertainty, which in turn leads to analytical processing. These results replicate and extend previous research on the ‘uncanny valley of mind’ (Kim et al., 2025), as they specify the underlying cognitive process through which inconsistent anthropomorphism leads to negative responses.

The dual-pathway conceptualization also helps reconcile contradictory findings in the anthropomorphism literature, which reports both facilitatory (enhanced trust and engagement) and inhibitory (uncanny valley and privacy concerns) effects. The DPCM attributes these to different processing pathways that are differentially engaged depending on context. The observed effect sizes (serial indirect effect = 0.18; interaction ηp^2^ = 0.06) match De Neys’s (2014) observation that dual-process effects tend to be small to medium in size, reflecting the complexity of interacting cognitive pathways, and align with broader accounts of reasoning errors and cognitive biases in judgment under uncertainty (Pennycook, 2023).

#### 5.2.2. Metacognitive Calibration as a Determinant of Decision Quality

These findings underscore the relatively unexplored, yet vitally important, function of metacognitive processes in human–AI interactions. In the past, various psychological studies have extensively investigated the concept of metacognition, particularly with regard to the context of academic and memory settings (Fleming, 2024). However, the specific concept of metacognition has not been explored with regard to its implications for the effective use of an AI system by an individual. The current study demonstrated that the link between AI reliance and decision-making quality was ultimately mediated through calibration, defined as an individual’s level of accuracy compared to their level of confidence.

This study extends the metacognition and confidence framework proposed by Fleming (2024), whose account holds that metacognitive monitoring lets people allocate cognitive resources efficiently and seek additional information when needed. The present results show that this adaptive function persists when the additional information comes from an AI system: individuals with well-calibrated metacognition can distinguish trustworthy from untrustworthy AI recommendations.

The moderation effect (β = 0.17) indicates that calibration explains meaningful variance in decision outcomes, consistent with Steyvers and Kumar’s (2024) discussion of the challenges of AI-supported decision-making. More importantly, the results suggest that miscalibration—whether expressed as algorithm aversion (Jussupow et al., 2020) or as uncritical acceptance—represents a metacognitive failure with practical consequences for judgment quality. This positions aversion not as an undifferentiated bias but as one facet of a broader calibration problem.

#### 5.2.3. Motivated Cognition and Pathway Activation

The findings of the research on the regulatory focus have implications for the understanding of the role of motivational states in social information processing from artificial agents. Regulatory Focus Theory (Werth & Foerster, 2007; Scholer et al., 2014) has shown that promotion and prevention orientation influence attention, memory, and evaluative responses. The research extends the theory by indicating that the regulation of focus influences the relative involvement of intuitive and analytical processing routes.

In particular, promotion-oriented individuals exhibited greater activation in the Social Closeness Pathway (ηp^2^ = 0.15), which implies that promotion orientation, with its emphasis on gains, facilitates the processing of affiliative information from anthropomorphic agents. Conversely, prevention-oriented individuals exhibited relatively greater activation in the Cognitive Evaluation Pathway (ηp^2^ = 0.05), which is consistent with their tendency to be vigilant and avoid errors. This asymmetry in the effect of regulatory focus, in which it mainly moderates Pathway A, implies that the affective and relational content of the Social Closeness Pathway is particularly sensitive to motivational effects.

The results build on the work of Scholer et al. (2014) on regulatory focus and risk-seeking by showing that the latter also affects the cognitive processing strategy employed in evaluating AI recommendations. The regulatory fit framework (Lee & Higgins, 2009) implies that information processing is more fluent when it fits the person’s current motivational orientation. The current results show that the latter also applies to the processing of social information provided by artificial agents.

#### 5.2.4. Nonlinear Dynamics in Social Perception of Artificial Agents

The inverted U-shape of the relationship between anthropomorphism and decision outcomes was significant (quadratic b = −0.05, *p* = 0.001), providing support for a non-monotonic function that was theoretically proposed but never before demonstrated in behavioral outcomes. Although the original Uncanny Valley Hypothesis was concerned with the perceived unpleasantness of near-human appearance (Song & Shin, 2024), the current research extends this principle to the field of judgment and decision-making.

The estimated optimum (approximately 4.6 on a seven-point scale) also bears on the cognitive representation of artificial agents. Low anthropomorphism may fail to trigger the social-cognitive processes essential for trust and engagement, whereas high anthropomorphism may generate expectancy violations that activate Pathway B and yield negative evaluations. A moderate level appears balanced, engaging social cognition without provoking the analytical scrutiny that undermines positive evaluations.

This nonlinear pattern is consistent with the psychophysiological findings of Ciechanowski et al. (2019) and extends those findings by providing behavioral consequences in relation to decision-making. Although this effect is small (ΔR^2^ = 0.03), it indicates that while this nonlinear pattern is supported, the level of anthropomorphism is but one of many factors involved in determining cognitive and behavioral outcomes—consistent with the concept of multidetermination of human judgment.

#### 5.2.5. Why the Effects Emerged: An Integrative Interpretation

Beyond confirming the hypothesized paths, the pattern of results invites an account of why these effects emerged and why they differed in magnitude. We suggest that anthropomorphic cues default to System 1 processing because they function as social affordances: humanlike language, naming, and conversational reciprocity activate person-perception schemas that the cognitive system is disposed to engage rapidly and with little deliberative cost (De Neys, 2014; Ciechanowski et al., 2019). On this reading, the affective pathway (Pathway A) is the more chronically accessible route, because relational and emotional cues are continuously available and require no triggering condition to operate.

This asymmetry helps explain why Pathway A was associated with a larger effect than the analytical pathway (Pathway B) (ηp^2^ = 0.15 vs. 0.05). Analytical override appears to be conditional: it is recruited mainly when an inconsistency or expectation violation signals that a more effortful check is warranted, so its contribution is engaged less often and operates on a narrower set of cases. The smaller estimate for Pathway B is therefore consistent with its proposed status as a corrective, demand-gated process rather than a constant input.

The moderation pattern is interpretable in the same terms. Regulatory focus moderated Pathway A more clearly than Pathway B, plausibly because affective and relational content is more motivation-sensitive than analytical evaluation: promotion- versus prevention-oriented goals shape how warmly relational cues are weighed, whereas analytic checking is comparatively goal-neutral (Scholer et al., 2014). The autonomy-by-reliance effect is most economically explained as reactance reduction: preserving perceived decision autonomy lowers resistance to the system’s input, allowing reliance to translate into adoption rather than provoking defensive discounting (Jussupow et al., 2020).

These interpretations also distinguish the DPCM from two narrower accounts. A pure CASA (computers are social actors) account anticipates the default social response we observe but does not, on its own, specify when analytical correction intervenes or why motivational orientation reshapes the affective route. A pure uncanny valley account predicts affective devaluation at high humanlikeness but cannot readily accommodate the calibration-gated, motivation-dependent structure of the present effects. We therefore offer the DPCM not as a replacement for these perspectives but as a broader integrative framework that situates both within a single dual-pathway architecture—a proposal that nonetheless awaits independent test.

### 5.3. Implications for Psychological Theory and Application

The theoretical contributions described above have implications not only for basic psychological science but also for applied fields that involve human–AI interaction.

Theoretically, the DPCM implies that dual-process models may need refinement for social cognition involving artificial agents. The System 1/System 2 distinction may not fully capture human–AI interaction, since anthropomorphic design can activate social processing while the agent’s artificial nature can simultaneously activate analytical processing. Future work may benefit from treating the anthropomorphism effect as the dynamic interplay of two or more cognitive systems rather than the activation of one or the other.

The metacognitive results suggest that individual differences in self-monitoring capacity may be particularly important in AI-augmented decision environments. As AI systems increasingly populate the environment in important domains, the cognitive factors that facilitate or hinder human–AI collaboration appear to be theoretically important. The present results suggest that metacognitive calibration should be considered more in models of human–AI interaction.

From an applied perspective, the effectiveness of an AI interface may be improved by attending to the cognitive processes the interface elicits. Interfaces that support user autonomy, provide appropriate transparency about AI capabilities, and avoid triggering expectancy violations through humanization are likely to be more effective in human–AI interaction. However, because the present studies did not examine real-world human–AI interaction and relied on scenario-based designs with a culturally homogeneous sample, these design considerations should be read as preliminary and theory-guided rather than prescriptive; recommendations such as adopting moderate rather than maximal anthropomorphism require validation in field settings before being applied (see Section 5.4).

### 5.4. Limitations and Future Directions

The present findings should be read in light of several limitations, each of which points to a specific direction for future work.

**Cultural homogeneity of the sample.** All participants were recruited in China, and anthropomorphic responding is shaped by culturally varying norms regarding social presence, politeness, and human–machine boundaries. The observed pathway structure, and particularly the relative weight of the affective route, may therefore not generalize to other cultural contexts. Future research should employ cross-cultural and ideally measurement-invariant designs to test whether the dual-pathway architecture and its calibration gate replicate across populations and to examine culture as a moderator of pathway strength.

**Common method bias.** Predictor and outcome constructs were assessed through a single source and the same self-report instrument, and largely on a single occasion, which raises the possibility of common-method variance inflating the observed associations. Procedural safeguards (e.g., separation of measurement sections and protection of respondent anonymity) were applied, and statistical checks are reported in Section 4.4; however, these remedies are partial. Future studies should incorporate multi-source and behavioral indicators, for example, observed choices, response latencies, or platform log data, and temporally separate the measurement of mediators and outcomes.

**Reliance on self-report.** Core constructs, including perceived warmth, trust, and metacognitive calibration, depend on participants’ introspective reports, which are susceptible to social desirability and limited introspective access, especially for fast, System 1-driven responses. Behavioral, physiological, or computational indicators would provide convergent evidence less dependent on introspection; process-tracing or neural correlates of the two pathways would help substantiate their proposed dissociation.

**Artificial settings and external validity.** The studies used scenario-based, simulated interactions rather than consequential decisions with real chatbots, which strengthens experimental control but constrains ecological validity. Participants did not bear genuine financial or relational stakes, and simulated agents omit the variability of deployed systems. Field experiments with live AI assistants, involving real choices and outcomes, are needed to establish whether the effects persist under naturalistic conditions.

**Cross-sectional measurement.** Although the manipulations were experimental, several mediating and moderating constructs were assessed cross-sectionally and concurrently with the outcomes, which limits inferences about temporal order and the causal direction of the mediational chain. Longitudinal and experience-sampling designs, and, where feasible, experimental manipulation of the proposed mediators such as calibration, would allow stronger temporal and causal claims.

**Generalizability and the uniformly supported hypothesis pattern.** The findings derive from a bounded set of consumer decision contexts, and all six hypotheses were supported; such a complete pattern, while encouraging, can also reflect researcher optimism, confirmation pressures, or sample- and method-specific features rather than the robustness of the model alone. The framework should therefore be tested across diverse AI applications and subjected to adversarial and pre-registered replication, together with explicit competing-model comparisons, so that the DPCM is evaluated as a falsifiable account rather than assumed.

Two further conceptual extensions follow from the present operationalizations. First, calibration was treated here as a measured individual difference; because it functions as the model’s corrective gate, a valuable next step is to manipulate it directly, through feedback, transparency, or confidence-prompting interventions, to test whether improving calibration causally strengthens analytical override. Second, anthropomorphism was modeled as a unitary construct, yet its visual, verbal, and behavioral facets may load differently onto the two pathways; decomposing anthropomorphism into these components would clarify which cues drive affective versus analytical processing.

## 6. Conclusions

Across three experiments, this research examined how AI chatbot anthropomorphism shapes consumer decision-making and found consistent evidence for two processing routes, an affective pathway and a conditional analytical pathway, whose influence depends on consumers’ metacognitive calibration and motivational orientation. The principal contribution is theoretical: the Dual-Pathway Calibration Model integrates dual-process, construal-level, metacognitive, and regulatory-focus perspectives into a single, falsifiable framework in which a calibration gate governs whether affective responses to humanlike cues are corrected. In doing so, the model moves beyond accounts that treat anthropomorphism as uniformly beneficial or uniformly unsettling. Practically, the findings caution against maximizing humanlikeness and instead favor moderate anthropomorphism paired with support for accurate user calibration, while recognizing the limited external validity of the present sample. We therefore offer the DPCM as a first integrative test rather than a settled conclusion, and we encourage replication across cultures, real interactions, and varied AI applications, alongside adversarial and competing-model evaluation, to establish the boundaries of the framework.

## Figures and Tables

**Table 1:** Summary of Research Hypotheses.

Hypothesis	Description	Theoretical Basis
**H1**	Chatbot anthropomorphism positively influences heuristic-based AI reliance through the serial mediation of reduced psychological distance and enhanced affective trust.	Construal Level Theory, CASA Paradigm
**H2**	The interaction between high anthropomorphism and cue inconsistency increases perceived uncertainty, which in turn activates analytical evaluation of AI recommendations.	Uncanny Valley of Mind, Expectancy Violation Theory
**H3**	Metacognitive calibration moderates the relationship between AI reliance and decision quality, such that this relationship is stronger when calibration is high.	Metacognition Theory
**H4a**	Promotion focus strengthens the activation of the Social Closeness Pathway (Pathway A).	Regulatory Focus Theory
**H4b**	Prevention focus strengthens the activation of the Cognitive Evaluation Pathway (Pathway B).	Regulatory Focus Theory
**H5**	Perceived autonomy moderates the relationship between AI reliance and decision satisfaction, such that this relationship is stronger when perceived autonomy is high.	Self-Determination Theory, Psychological Reactance Theory
**H6**	Chatbot anthropomorphism exhibits an inverted U-shaped relationship with consumer decision outcomes (Exploratory).	Dual-Pathway Integration

**Table 2:** Overview of Experimental Studies.

Study	Sample Size	Design	Context	Focal Hypotheses
**1**	N = 286	3 (Anthropomorphism) × 2 (Cue Inconsistency)	Financial product recommendation	H1, H2
**2**	N = 234	2 (Anthropomorphism) × 2 (Regulatory Focus) × 2 (Cue Inconsistency; secondary factor)	Health product recommendation	H3, H4a, H4b; Pathway B supplementary
**3**	N = 312	3 (Anthropomorphism) × 2 (Perceived Autonomy)	Online shopping assistance	H5, H6

**Table 3:** Anthropomorphism Manipulation Specifications.

Dimension	Low	Medium	High
**Visual appearance**	Abstract AI icon	Cartoon avatar	Realistic photograph
**Identity**	System code	Friendly nickname	Personal name with title
**Language style**	Formal, technical	Conversational	Warm, empathetic
**Emotional expression**	Absent	Minimal	Frequent
**Self-disclosure**	Absent	Absent	Present

**Table 4:** Descriptive Statistics and Correlations (Study 1, N = 286).

Variable	M	SD	1	2	3	4	5	6	7	8
**1. Perceived anthropomorphism**	4.12	1.54	(0.92)							
**2. Psychological distance**	3.67	1.21	−0.38 ***	(0.89)						
**3. Affective trust**	4.28	1.18	0.41 ***	−0.52 ***	(0.91)					
**4. Heuristic reliance**	3.89	1.24	0.35 ***	−0.41 ***	0.58 ***	(0.85)				
**5. Perceived uncertainty**	3.54	1.31	−0.12 *	0.29 ***	−0.34 ***	−0.31 ***	(0.88)			
**6. Analytical evaluation**	4.21	1.15	−0.08	0.22 ***	−0.19 **	−0.27 ***	0.46 ***	(0.84)		
**7. Decision quality**	68.42	15.73	0.24 ***	−0.21 ***	0.31 ***	0.18 **	−0.15 *	0.11	—	
**8. Decision satisfaction**	4.56	1.08	0.37 ***	−0.44 ***	0.61 ***	0.52 ***	−0.28 ***	−0.14 *	0.39 ***	(0.90)

Note. Cronbach’s α coefficients are shown in parentheses on the diagonal. * *p* < 0.05, ** *p* < 0.01, *** *p* < 0.001.

**Table 5:** Serial Mediation Results for H1 (PROCESS Model 6).

Path	Effect	SE	95% CI	*p*
**High vs. Low Anthropomorphism**				
**Total effect (c)**	0.73	0.13	[0.48, 0.98]	<0.001
**Direct effect (c** **′** **)**	0.31	0.12	[0.07, 0.55]	0.012
**Total indirect effect**	0.42	0.08	[0.27, 0.58]	—
**Indirect via PD only**	0.14	0.04	[0.06, 0.23]	—
**Indirect via AT only**	0.09	0.03	[0.04, 0.16]	—
**Serial indirect (PD → AT)**	0.19	0.05	[0.11, 0.29]	—
**Medium vs. Low Anthropomorphism**				
**Total effect (c)**	0.38	0.13	[0.13, 0.63]	0.003
**Direct effect (c** **′** **)**	0.19	0.12	[−0.05, 0.43]	0.118
**Total indirect effect**	0.19	0.05	[0.10, 0.30]	—
**Serial indirect (PD → AT)**	0.09	0.03	[0.04, 0.16]	—

Note. PD = psychological distance; AT = affective trust. Bootstrap 95% confidence intervals based on 5000 resamples.

**Table 6:** ANOVA Results for H2 Testing.

Source	df	Perceived Uncertainty		Analytical Evaluation	
		F	ηp^2^	F	ηp^2^
**Anthropomorphism (A)**	2	2.17	0.02	1.84	0.01
**Cue Inconsistency (CI)**	1	78.42 ***	0.22	31.27 ***	0.10
**A × CI**	2	8.93 ***	0.06	5.62 **	0.04
**Error**	280				

Note. ** *p* < 0.01, *** *p* < 0.001.

**Table 7:** Descriptive Statistics and Correlations (Study 2, N = 234).

Variable	M	SD	1	2	3	4	5	6	7	8
**1. Perceived anthropomorphism**	4.08	1.67	(0.93)							
**2. AI reliance**	4.14	1.22	0.39 ***	(0.87)						
**3. Metacognitive calibration**	0.41	0.26	0.06	0.08	—					
**4. Pathway activation index**	0.23	1.08	0.44 ***	0.51 ***	−0.11	—				
**5. Heuristic reliance**	3.94	1.19	0.38 ***	0.67 ***	−0.04	0.51 ***	(0.86)			
**6. Analytical evaluation**	4.17	1.12	−0.11	−0.24 ***	0.18 **	−0.47 ***	−0.29 ***	(0.83)		
**7. Decision quality**	65.87	16.24	0.18 **	0.22 ***	0.34 ***	0.09	0.12	0.21 **	—	
**8. Decision satisfaction**	4.48	1.14	0.41 ***	0.58 ***	0.14 *	0.43 ***	0.54 ***	−0.16 *	0.36 ***	(0.91)

Note. Metacognitive calibration = reverse-scored Brier Score (higher values indicate better calibration). Pathway activation index = Pathway A processing score minus Pathway B processing score (positive values indicate greater Pathway A activation). Cronbach’s α coefficients shown in parentheses. * *p* < 0.05, ** *p* < 0.01, *** *p* < 0.001.

**Table 8:** Moderated Regression Results for H3.

Predictor	Step 1		Step 2		Step 3	
	β	*p*	β	*p*	β	*p*
**Control Variables**						
**Age**	0.04	0.562	0.02	0.731	0.02	0.758
**Gender**	−0.06	0.384	−0.05	0.421	−0.04	0.487
**Education**	0.11	0.097	0.08	0.198	0.07	0.241
**Prior AI experience**	0.09	0.178	0.05	0.438	0.05	0.451
**Need for cognition**	0.14 *	0.038	0.09	0.152	0.08	0.187
**Main Effects**						
**AI reliance (AIR)**			0.19 **	0.004	0.18 **	0.006
**Metacognitive calibration (MC)**			0.31 ***	<0.001	0.30 ***	<0.001
**Interaction**						
**AIR × MC**					0.17 **	0.007
**R^2^**	0.05		0.18		0.21	
**ΔR^2^**			0.13 ***		0.03 **	
**F**	2.34 *		7.18 ***		7.42 ***	

Note. N = 234. Standardized coefficients reported. * *p* < 0.05, ** *p* < 0.01, *** *p* < 0.001.

**Table 9:** ANOVA Results for H4 Testing.

Source	df	SS	MS	F	*p*	ηp^2^
**Anthropomorphism (A)**	1	45.18	45.18	45.72	<0.001	0.17
**Regulatory Focus (RF)**	1	28.01	28.01	28.34	<0.001	0.11
**A × RF**	1	12.52	12.52	12.67	<0.001	0.05
**Error**	230	227.24	0.99			
**Total**	233					

Note. Dependent variable = Pathway activation index.

**Table 10:** Descriptive Statistics and Correlations (Study 3, N = 312).

Variable	M	SD	1	2	3	4	5	6	7
**1. Perceived anthropomorphism**	4.15	1.58	(0.94)						
**2. Perceived autonomy**	4.42	1.34	0.08	(0.89)					
**3. Psychological reactance**	3.21	1.27	0.05	−0.54 ***	(0.86)				
**4. AI reliance**	4.08	1.19	0.36 ***	0.31 ***	−0.28 ***	(0.85)			
**5. Heuristic reliance**	3.91	1.21	0.34 ***	0.27 ***	−0.24 ***	0.64 ***	(0.87)		
**6. Decision quality**	67.24	15.18	0.19 **	0.23 ***	−0.17 **	0.25 ***	0.16 **	—	
**7. Decision satisfaction**	4.51	1.11	0.32 ***	0.47 ***	−0.41 ***	0.54 ***	0.49 ***	0.38 ***	(0.92)

Note. Cronbach’s α coefficients shown in parentheses. ** *p* < 0.01, *** *p* < 0.001.

**Table 11:** Moderated Regression Results for H5.

Predictor	Step 1		Step 2		Step 3	
	β	*p*	β	*p*	β	*p*
**Control Variables**						
**Age**	0.02	0.714	0.01	0.842	0.01	0.867
**Gender**	0.05	0.367	0.04	0.438	0.03	0.512
**Education**	0.08	0.164	0.05	0.324	0.05	0.341
**Prior AI experience**	0.12 *	0.034	0.07	0.178	0.06	0.214
**Need for cognition**	0.06	0.287	0.03	0.541	0.03	0.558
**Main Effects**						
**AI reliance (AIR)**			0.41 ***	<0.001	0.39 ***	<0.001
**Perceived autonomy (PA)**			0.34 ***	<0.001	0.33 ***	<0.001
**Interaction**						
**AIR × PA**					0.14 **	0.008
**R^2^**	0.04		0.35		0.37	
**ΔR^2^**			0.31 ***		0.02 **	
**F**	2.41 *		23.14 ***		21.87 ***	

Note. N = 312. Standardized coefficients reported. * *p* < 0.05, ** *p* < 0.01, *** *p* < 0.001.

**Table 12:** Polynomial Regression Results for H6.

	Decision Satisfaction		Decision Quality	
**Predictor**	b (SE)	*p*	b (SE)	*p*
**Step 1: Controls**				
**R^2^**	0.04		0.05	
**Step 2: Linear**				
**Anthropomorphism**	0.23 (0.04)	<0.001	2.14 (0.58)	<0.001
**ΔR^2^**	0.10 ***		0.06 ***	
**Step 3: Quadratic**				
**Anthropomorphism**	0.41 (0.11)	<0.001	4.87 (1.52)	0.002
**Anthropomorphism^2^**	−0.05 (0.01)	0.001	−0.71 (0.22)	0.001
**ΔR^2^**	0.03 **		0.02 **	
**Total R^2^**	0.17		0.13	
**Inflection point**	4.62		4.31	

Note. N = 312. Unstandardized coefficients reported. ** *p* < 0.01, *** *p* < 0.001.

**Table 13:** Summary of Hypothesis Testing Across Three Studies.

Hypothesis	Study/Analysis Sample	Key Statistic	Effect Size	95% CI	Supported
**H1: Serial mediation (PD → AT)**	1	Indirect effect	0.19	[0.11, 0.29]	Yes
	3	Indirect effect	0.17	[0.10, 0.26]	Yes
	Studies 1 and 3	Indirect effect	0.18	[0.13, 0.24]	Yes
**H2: A × CI interaction**	1	ηp^2^	0.06	—	Yes
	Studies 1 and 2	ηp^2^	0.05	—	Yes
**H3: Calibration moderation**	2	β	0.17	—	Yes
	3	β	0.19	—	Yes
	Study 2 and Study 3 subset	β	0.15	—	Yes
**H4a: Promotion → Pathway A**	2	ηp^2^	0.15	—	Yes
**H4b: Prevention → Pathway B**	2	ηp^2^	0.05	—	Yes
**H5: Autonomy moderation**	3	β	0.14	—	Yes
	Study 3	β	0.12	—	Yes
**H6: Inverted U-shape**	3	Quadratic b	−0.05	—	Yes
	Studies 1–3	Quadratic b	−0.04	—	Yes

Note. A = Anthropomorphism; CI = Cue Inconsistency; PD = Psychological Distance; AT = Affective Trust.

**Table 14:** Summary of Hypothesis Testing Results.

Hypothesis	Prediction	Study	Result	Key Statistics
**H1**	Anthropomorphism → Psychological Distance↓ → Affective Trust↑ → Heuristic Reliance↑ (Serial Mediation)	1	Supported	Indirect effect = 0.19, 95% CI [0.11, 0.29]
		3	Supported	Indirect effect = 0.17, 95% CI [0.10, 0.26]
**H2**	High Anthropomorphism × Cue Inconsistency → Uncertainty↑ → Analytical Evaluation↑	1	Supported	F(2, 280) = 8.93, *p* < 0.001, ηp^2^ = 0.06
**H3**	Metacognitive Calibration moderates AI Reliance → Decision Quality	2	Supported	β = 0.17, *p* = 0.007
		3	Supported	β = 0.19, *p* = 0.018
**H4a**	Promotion Focus strengthens Pathway A	2	Supported	Simple effect: F(1, 230) = 41.28, *p* < 0.001, ηp^2^ = 0.15
**H4b**	Prevention Focus strengthens Pathway B	2	Supported	Simple effect: F(1, 230) = 11.54, *p* < 0.001, ηp^2^ = 0.05
**H5**	Perceived Autonomy moderates AI Reliance → Decision Satisfaction	3	Supported	β = 0.14, *p* = 0.008
**H6**	Inverted U-shaped relationship between Anthropomorphism and Decision Outcomes	3	Supported	Quadratic b = −0.05, *p* = 0.001; Optimal = 4.62

**Table 15:** Effect Size Summary and Interpretation.

Effect	Effect Size	Metric	Interpretation
**Anthropomorphism → Psychological Distance**	−0.87	b	Large
**Psychological Distance → Affective Trust**	−0.41	b	Medium
**Affective Trust → Heuristic Reliance**	0.53	b	Large
**Serial Indirect Effect (H1)**	0.18	Indirect	Medium
**Anthropomorphism × Cue Inconsistency (H2)**	0.06	ηp^2^	Medium
**Calibration Moderation (H3)**	0.17	β	Small-Medium
**Regulatory Focus × Anthropomorphism (H4)**	0.05	ηp^2^	Small-Medium
**Autonomy Moderation (H5)**	0.14	β	Small-Medium
**Quadratic Effect (H6)**	0.03	ΔR^2^	Small

Note. Effect size interpretation based on Cohen’s conventions: small (ηp^2^ = 0.01, β = 0.10), medium (ηp^2^ = 0.06, β = 0.30), and large (ηp^2^ = 0.14, β = 0.50).

## Data Availability

The data presented in this study are available upon request from the corresponding author.

## Appendix A. Measurement Scales and Items

All multi-item constructs were measured on seven-point Likert scales (1 = strongly disagree, 7 = strongly agree) unless otherwise noted. Established scales were administered in Chinese following back-translation procedures; the English wordings are presented below. (R) denotes reverse-scored items. Table A1 lists the focal measures used in the mediation and outcome analyses, and Table A2 lists the moderator, manipulation-check, and control measures.

**Table A1:** Focal Measurement Items (Studies 1–3).

Construct (Source; Reliability)	Item	Wording
**Perceived anthropomorphism (manipulation check; adapted from Bartneck et al.; α = 0.92)**	PA1	The chatbot seemed natural rather than fake.
	PA2	The chatbot seemed humanlike rather than machinelike.
	PA3	The chatbot seemed conscious rather than unconscious.
	PA4	The chatbot seemed lifelike rather than artificial.
**Perceived cue inconsistency (manipulation check; α = 0.87)**	CI1	Some of the chatbot’s responses were inconsistent with what it had said earlier.
	CI2	The chatbot’s recommendation did not fully match the preferences I had stated.
	CI3	Some of the chatbot’s responses did not fit the context of our conversation.
**Psychological distance (adapted from Kim et al.; α = 0.89)**	PD1	The chatbot felt distant to me.
	PD2	Interacting with the chatbot felt impersonal.
	PD3	I felt a sense of closeness to the chatbot. (R)
	PD4	The chatbot felt psychologically near to me. (R)
**Affective trust (adapted from McAllister; α = 0.91)**	AT1	I felt comfortable sharing my needs and concerns with the chatbot.
	AT2	I felt that the chatbot genuinely cared about my interests.
	AT3	I felt an emotional bond of trust toward the chatbot.
	AT4	If I could no longer use this chatbot, I would feel a sense of loss.
**Heuristic reliance (α = 0.85)**	HR1	I accepted the chatbot’s recommendation without thinking much about it.
	HR2	I went with my gut feeling when responding to the chatbot’s advice.
	HR3	I relied on my first impression of the recommendation.
	HR4	I did not find it necessary to examine the recommendation in detail.
**Perceived uncertainty (adapted from Milliken; α = 0.88)**	PU1	I was unsure whether the chatbot’s recommendation could be trusted.
	PU2	I found it difficult to predict how accurate the chatbot’s advice was.
	PU3	I felt uncertain about the quality of the information the chatbot provided.
	PU4	I could not confidently judge the reliability of the chatbot’s advice.
**Analytical evaluation (α = 0.84)**	AE1	I carefully examined the reasons behind the chatbot’s recommendation.
	AE2	I compared the recommendation against other information available to me.
	AE3	I critically evaluated whether the recommendation fit my situation.
	AE4	I deliberated thoroughly before accepting or rejecting the advice.
**Decision confidence (α = 0.83)**	DC1	I am confident that I made a good decision.
	DC2	I am certain that my choice was the right one for me.
	DC3	If I faced the same situation again, I would make the same decision.
**Decision satisfaction (adapted from Fitzsimons; α = 0.90)**	DS1	I am satisfied with my final decision.
	DS2	The decision process left me with a positive feeling.
	DS3	I am pleased with the option I chose.
	DS4	Overall, this was a satisfying decision experience.

**Table A2:** Moderator, Manipulation-Check, and Control Measures.

Construct (Source; Reliability)	Item	Wording
**Promotion focus, situational (manipulation check, Study 2; α = 0.85)**	PRO1	Right now, I am focused on achieving positive outcomes for my health.
	PRO2	At this moment, I am thinking about my hopes and aspirations.
	PRO3	I am currently oriented toward attaining gains.
**Prevention focus, situational (manipulation check, Study 2; α = 0.84)**	PRE1	Right now, I am focused on avoiding negative outcomes for my health.
	PRE2	At this moment, I am thinking about my duties and responsibilities.
	PRE3	I am currently oriented toward preventing losses.
**Pathway activation—social–emotional processing (Study 2; α = 0.83)**	SE1	I responded to the chatbot mainly on the basis of my feelings.
	SE2	I paid attention to the social and emotional aspects of the conversation.
	SE3	My reactions to the chatbot were spontaneous rather than deliberate.
**Pathway activation—analytical processing (Study 2; α = 0.81)**	AN1	I processed the chatbot’s information in a step-by-step, logical manner.
	AN2	I focused on the factual content of the recommendation rather than on its style.
	AN3	I systematically weighed the pros and cons of the advice.
**Perceived autonomy (Study 3; α = 0.89)**	AU1	I felt free to make my own choice.
	AU2	The chatbot respected my ability to decide for myself.
	AU3	I felt in control of the final decision.
	AU4	I could easily deviate from the chatbot’s recommendation.
	AU5	The final choice reflected my own preferences.
**Psychological reactance (Study 3; α = 0.86)**	RE1	I felt pressured to accept the chatbot’s recommendation.
	RE2	I felt that the chatbot was trying to make the decision for me.
	RE3	I felt that my freedom of choice was restricted.
	RE4	I wanted to resist the chatbot’s suggestions.
**Trust propensity (control; α = 0.79)**	TP1	Most people can be trusted.
	TP2	I tend to trust new technologies until proven otherwise.
	TP3	I generally trust recommendations from automated systems.
**Need for cognition (control, short form; α = 0.82)**	NC1	I enjoy tasks that require a lot of thinking.
	NC2	I prefer complex problems to simple ones.
	NC3	Thinking hard about something for a long time gives me satisfaction.
**Financial literacy (control, Study 1; 4-item quiz, number correct 0–4)**	FL1	Compound interest: growth of savings over five years at 2% annual interest (multiple choice).
	FL2	Inflation: purchasing power after one year when inflation exceeds the interest rate (multiple choice).
	FL3	Diversification: whether buying a single company stock is safer than a stock mutual fund (true/false).
	FL4	Risk–return: whether higher expected returns generally entail higher risk (true/false).

Note. Objective decision quality was scored as 0–100 proximity to the expert benchmark described in Section 3.2.4 and Appendix C; it was not a self-report scale.

## Appendix B. Experimental Manipulation Materials

Table A3 summarizes the anthropomorphism manipulation scripts implemented in the chatbot interface (Studies 1 and 3 used all three levels; Study 2 used the low and high levels). Table A4 summarizes the operationalization of the cue-inconsistency manipulation. Full-color screenshots of every condition, the complete dialogue scripts, and the exact Chinese wordings are archived on the OSF.

**Table A3:** Anthropomorphism Manipulation Scripts (Examples from the Study 1 Financial Advisory Context).

Design Element	Low	Medium	High
**Visual appearance**	Abstract geometric AI icon	Stylized cartoon avatar	Realistic human photograph
**Identity label**	“Advisory System A-7”	“Xiaozhi” (friendly nickname)	“Advisor Li Wei” (personal name with title)
**Greeting (example)**	“System ready. Please state your investment parameters.”	“Hi! I’m Xiaozhi. Happy to help you find a good option today!”	“Hello, I’m Li Wei, your personal advisor. Decisions like this can feel stressful—I’ll walk through it with you step by step.”
**Recommendation phrasing (example)**	“Based on the entered parameters, Option B satisfies the stated constraints.”	“Looking at what you told me, I think Option B could be a good fit for you.”	“I understand what matters to you, and I truly think Option B suits your goals. I would feel comfortable choosing it myself.”
**Emotional expression**	Absent	Minimal (occasional friendly phrases)	Frequent (empathy, reassurance)
**Self-disclosure**	Absent	Absent	Present (e.g., “I remember how it felt when I faced my first decision like this.”)

**Table A4:** Operationalization of the Cue-Inconsistency Manipulation.

Cue Type	Implementation
**Preference-mismatched recommendation**	A recommendation partially inconsistent with the risk preference (Study 1), health goal (Study 2), or feature priorities (Study 3) that the participant had stated earlier in the dialogue.
**Repeated question**	The chatbot asked again a question that the participant had already answered (e.g., asking for the budget range a second time).
**Contextually mismatched response**	A reply that did not fit the immediately preceding user input (e.g., answering about fees when the participant asked about risk).

**Consistent-cue conditions.** In the consistent-cue conditions, the three cues were omitted while interface length, wording, and recommendation valence were held constant.

**Regulatory-focus induction (Study 2).** Participants in the promotion condition wrote for three minutes about their health aspirations and the ideal outcomes they hoped to attain; participants in the prevention condition wrote about their health responsibilities and the negative outcomes they sought to avoid.

**Perceived-autonomy manipulation (Study 3).** In the high-autonomy condition, recommendations used suggestive language (“you might consider…”), alternatives were always displayed, the recommendation could be modified in one step, and autonomy-supporting statements (“the choice is entirely yours”) were included. In the low-autonomy condition, recommendations were assertive (“the best option for you is…”), alternatives were limited, the recommended option was preselected as the default, and statements emphasized the superiority of the chatbot’s judgment.

## Appendix C. Metacognitive Calibration Task and Expert Benchmark

**Calibration task.** Participants answered eight factual multiple-choice questions about the attributes of the options that the chatbot had presented (e.g., fees, risk level, historical performance, minimum investment in Study 1; ingredients, dosage, and certification in Study 2; specifications and price in Study 3). Each answer was followed by a confidence rating on a 50–100% scale. Calibration was indexed by the Brier score, BS = (1/*n*) × Σ (ci − oi)^2^, where ci is the stated confidence (rescaled to 0–1) and oi the binary accuracy of item i; lower scores indicate better calibration. The task was administered immediately after the decision task and before debriefing.

**Sample item.** Sample item (Study 1): “According to the chatbot, which of the four funds had the lowest management fee?” (four response options), followed by “How confident are you in your answer?” (50–100%). The full question sets for all three studies are archived on the OSF.

**Expert benchmark.** As detailed in Section 3.2.4, objective decision quality was scored as the 0–100 proximity of the chosen option to the option(s) identified as normatively optimal by independent, choice-blind expert panels (certified financial planners in Study 1; registered dietitians and qualified health professionals in Study 2; consumer-electronics specialists in Study 3). Inter-rater agreement ranged from ICC = 0.84 to 0.91. The scoring protocols are archived on the OSF.
